# Proteomic Profiling of Primary Hippocampal Neurons Reveals Noncanonical GFAP Expression and Metabolic Adaptations in Glia‐Free Culture

**DOI:** 10.1002/pmic.70130

**Published:** 2026-04-10

**Authors:** Dominika Drulis‐Fajdasz, Kinga Gostomska‐Pampuch, Natalia Pudełko‐Malik, Mariusz Fleszar, Agnieszka Gizak, Jacek R. Wiśniewski, Dariusz Rakus

**Affiliations:** ^1^ Department of Molecular Physiology and Neurobiology University of Wroclaw Wroclaw Poland; ^2^ Department of Biochemistry and Immunochemistry Wroclaw Medical University Wroclaw Poland; ^3^ Department of Biochemistry Molecular Biology and Biotechnology, Faculty of Chemistry Wroclaw University of Science and Technology Wroclaw Poland; ^4^ Omics Research Center Wroclaw Medical University Wroclaw Poland; ^5^ Biochemical Proteomics Group Department of Proteomics and Signal Transduction Max Planck Institute of Biochemistry Martinsried Germany

**Keywords:** astrocyte and neuron markers, energy metabolism, synaptic transmission, total protein approach

## Abstract

Despite their widespread use as a research model, a comprehensive, quantitative proteomic profile of the cultured hippocampal neurons has remained unexplored. Here, we provide the first global proteomic characterization of primary murine hippocampal neurons cultured for 14 days under near‐physiological glucose conditions (2.5 mM). Using label‐free mass spectrometry and the Total Protein Approach (TPA), we quantified over 5,500 proteins, revealing a metabolic signature consistent with oxidative phosphorylation and a neuron‐specific proteome largely devoid of astrocytic markers. Unexpectedly, glial fibrillary acidic protein (GFAP) was highly abundant in pure neuronal cultures, and its knockdown impaired neuronal viability and morphology. These findings suggest a compensatory role for GFAP in neurons cultured without astrocytic support. Additionally, our data highlight significant underexpression of key synaptic proteins compared to hippocampal tissue, underscoring the limitations of neuron‐only culture systems. This study establishes a new molecular reference for hippocampal neurons and offers insights into neuronal adaptation in simplified environments.

**Abbreviations**
ANCC: astrocyte–neuron co‐cultures
cLTD: chemical long‐term depression
cLTP: chemical long‐term potentiation
copGFP: green fluorescent protein from copepod *Pontellina* sp
GFAP: glial fibrillary acidic protein
MAP2: microtubule‐associated protein 2
NC: in neuronal monocultures
NeuN: Fox‐3, Rbfox3, or Hexaribonucleotide Binding Protein‐3 (a biomarker for neurons)
OXPHOS: oxidative phosphorylation
TCA cycle: tricarboxylic acid cycle
TPA: total protein approach

## 1 Introduction

The hippocampus is a central structure for learning and memory, in which long‐lasting changes in synaptic efficacy (e.g., long‐term potentiation and long‐term depression) are coupled to coordinated remodeling of receptor composition and density, intracellular signaling pathways, cytoskeletal organization, and membrane trafficking [1]. These processes impose substantial energetic demands on neurons and require tight integration of synaptic activity with metabolic capacity and proteostasis. In vitro hippocampal neurons cultures offer experimental accessibility and control over activity‐dependent variables, enabling mechanistic studies of neuronal growth, synaptogenesis, and plasticity. At the same time, neuronal physiology in culture depend on the extracellular milieu and the extent of neuron–glia interactions, underscoring the value of well‐defined baseline molecular references for specific culture paradigms.

Despite the widespread use of these cultures in molecular and cellular studies, a comprehensive, quantitative description of the hippocampal neuronal proteome remains lacking. Previous studies have focused either on specific proteins or protein classes, or analyzed subcellular fractions such as synaptosomes [2, 3] but to our knowledge, no global quantitative proteomic characterization of cultured mouse hippocampal neurons has yet been reported. A quantitative reference is important because transcript abundance does not necessarily predict protein abundance, and because the functional capacity of pathways critical for neuronal signaling and plasticity—such as synaptic transmission, calcium handling, mitochondrial respiration, and cytoskeletal dynamics—is ultimately constrained by protein copy numbers and concentrations.

In the present study, we employed a mass spectrometry–based label‐free quantitative proteomic method—the Total Protein Approach (TPA)—to provide an in‐depth analysis of the proteome of primary murine hippocampal neurons [4]. TPA enables estimation of protein abundances in a manner that supports quantitative comparisons across pathways and functional modules. Cultures were grown under near‐physiological conditions, including the use of a low‐glucose medium (2.5 mM), a concentration comparable to that found in the cerebrospinal fluid in vivo [5, 6]. Neuronal purity exceeded 95%, as confirmed by immunostaining, and neuronal functionality was validated using established chemical paradigms of synaptic potentiation and depression.

Using this approach, we generated a quantitative proteomic resource for primary hippocampal neurons, quantifying over 5,500 proteins. This dataset is intended to serve as a reference for the proteomic landscape of hippocampal neurons maintained under defined, near‐physiological culture conditions, and to support hypothesis generation regarding pathway allocation between synaptic, structural, and metabolic systems.

During quality‐control and cell‐type marker assessment, most astrocytic markers were not detected in neuronal cultures; however, glial fibrillary acidic protein (GFAP) was detected at levels that warranted further examination. This observation motivated orthogonal validation and downstream experiments aimed at clarifying the cellular source of GFAP signal in astrocyte‐limited preparations and exploring potential functional implications under these culture conditions.

Finally, we used the quantitative proteome to examine the representation of proteins associated with core metabolic pathways (including glycolysis, the TCA cycle, and oxidative phosphorylation) and with synaptic transmission and plasticity‐related signaling, and we compared the neuronal culture proteome to hippocampal tissue from juvenile mice. Together, these analyses provide a framework for contextualizing neuron‐intrinsic proteomic features in culture relative to tissue composition and for informing future studies of neuronal identity, function, and plasticity.

## 2 Materials and Methods

### 2.1 Animal Treatment

Animals were obtained from the Experimental Animal Facility of the Medical University of Wroclaw. The experiments were performed using female C57BL/10J mice (P30, *N*  =  5). Animals were treated as we described before [7] they were anesthetized with isoflurane and then decapitated. The protocol complied with standards of EU Directive 2010/63/EU for animal experiments and was approved by the II Local Scientific Research Ethical Committee, Wroclaw University of Environmental and Life Sciences, and the study is reported in accordance with ARRIVE guidelines.

### 2.2 Preparation of Tissue Lysates

Hippocampi were isolated in an ice‐cold buffer containing: 87 mM NaCl, 2.5 mM KCl, 1.25 mM NaH_2_PO_4_, 25 mM NaHCO_3_, 0.5 mM CaCl_2_, 7 mM MgSO_4_, 25 mM glucose, and 75 mM sucrose; pH 7.4. Immediately after isolation, the right hippocampus from each animal was homogenized in the buffer containing 0.1 M Tris‐HCl (pH 8.0), 2% SDS, 50 mM DTT, followed by incubation at 99°C for 5 min. The samples were subsequently snap‐frozen in liquid nitrogen and stored at ‐80°C until proteomic analysis. Total protein concentration was determined by measuring tryptophan fluorescence as previously described [8]. Briefly, the measurements were carried out in 96‐well white flat‐bottomed polystyrene plate. 1 µL of tissue lysate were spiked in 200 µL of buffered urea (8 M Urea, 100 mM Tris‐HCl pH 8.5, 1 mM DTT). Tryptophan were used in concentration of 0.1 µg/µL. Emission spectra of tryptophan were recorded using Infinite 200 (Tecan) plate reader. The excitation was set to 295 nm with a 5 nm bandwidth and the emission to 350 nm with a 20 nm bandwidth. Individual measurements consisted of 10 reads each with 50 µs integration time.

### 2.3 Cell Culture

#### 2.3.1 Primary Neuronal Culture

Primary hippocampal neurons were obtained from 0–2 day old C57BL6 mice derived from a single litter. Tissue dissection was performed on ice using a dissection medium (DM) composed of: 81.8 mM Na_2_SO_4_, 30 mM K_2_SO_4_, 5.8 mM MgCl_2_, 0.25 mM CaCl_2_, 1 mM HEPES, 0.2 mM NaOH, and 20 mM glucose; pH 7.4. After isolation, the DM was removed, and the tissue was enzymatically dissociated by incubation at 37°C with 0.25% w/v trypsin in 0.02% EDTA prepared in DM. The digestion was carried out in two steps: initially at a 1:2 trypsin‐to‐DM ratio for 15 min, followed by a 2:1 ratio for an additional 10 min. After that, MEM‐FBS (Minimal Essential Medium with the Fetal Bovine Serum) (BioWest, Bourg, France) medium was added to stop trypsin activity. The MEM‐FBS medium was then removed, and the tissue was gently triturated in fresh MEM‐FBS. The resulting cell suspension was centrifuged at 234 × g for 5 min at 25°C. After removing the supernatant, the cell pellet was resuspended in MEM‐FBS. Glass coverslips were coated with the coating solution (borate buffer: 80 mM H_3_BO_3_, 20 mM Na_2_B_4_O_7_, 2.5 µg/mL laminin, 0.1 mg/mL poly‐L‐lysine) and rinsed thoroughly before cells were plated at density of 25,000 cells/cm^2^. Two hours post‐plating, the MEM‐FBS medium was replaced with a neuronal medium consisting of: Neurobasal A (ThermoFisher Scientific, Waltham, MA, USA), 2% B27 Supplement (ThermoFisher Scientific, Waltham, MA, USA), 0.5 mM glutamine (Merck KGaA, Darmstadt, Germany), 12.5 µM glutamate (Merck KGaA, Darmstadt, Germany), and 1% penicillin/streptomycin (BioWest, Bourg, France), and 2.5 mM glucose. To ensure the purity of the neuronal monoculture, 10 µM cytosine β‐D‐arabinofuranoside (Ara‐C; Merck KGaA, Darmstadt, Germany) a mitotic inhibitor eliminating proliferating nonneuronal cells, was added 24 h after plating. Cells were maintained at 37°C in a humidified atmosphere containing 5% CO_2_. The experiments were performed on 4‐14‐day‐old neuronal monocultures.

#### 2.3.2 Astrocytic Cultures

Primary astrocyte cultures were established from neonatal from 0–2 day old C57BL6 mice and cultured using a modified protocol [9]. Briefly, hippocampal tissue was dissected from newborn animals, mechanically dissociated, and seeded at a density of 25,000 cells/cm^2^ (onto glass coverslips coated in the same manner as those used for primary neuronal cultures) in Dulbecco's Modified Eagle Medium (DMEM) (Biowest, low glucose, Bourg, France) containing 5 mM glucose and 0.11 g/L sodium pyruvate. The culture medium was supplemented with 2 mM L‐glutamine (Merck KGaA, Darmstadt, Germany), 10% fetal bovine serum (FBS) (BioWest, Bourg, France), 100 U/mL penicillin (BioWest, Bourg, France), and 0.1 mg/mL streptomycin (BioWest, Bourg, France). To suppress fibroblast proliferation, L‐valine was replaced with 0.094 g/L D‐valine (Merck KGaA, Darmstadt, Germany). The experiments were performed on 14‐day‐old astrocyte monocultures.

#### 2.3.3 Astrocyte‐Neuron Co‐Cultures

To establish an astrocyte‐neuron co‐culture model, an analogous procedure was applied as described for primary neuronal cultures, with the exception that cytosine β‐D‐arabinofuranoside (Ara‐C) was omitted. The experiments were performed at day 7 and 14 for astrocyte‐neuronal co‐cultures.

### 2.4 Preparation of Neuronal Lysates and Nuclear Fraction Isolation

After 14 days in culture neurons were fractionated using the Rapid Efficient And Practical (REAP) nuclear/cytoplasmic separation protocol, as described by Suzuki et al. [10]. Briefly, cells were washed with ice‐cold phosphate‐buffered saline (PBS, pH 7.4), scraped from the culture dishes on ice using a plastic cell scraper, and transferred into 1.5 mL microcentrifuge tubes containing 1 mL of ice‐cold PBS. Following a brief centrifugation (“pop‐spin”) for 10 s in a tabletop microcentrifuge (MPW‐260R), the supernatant was discarded, and the cell pellet was resuspended in 900 µL of ice‐cold 0.1% NP‐40 (74385, Fluka, Seelze, Germany) in PBS. From this lysate, 300 µL was collected as the whole cell lysate. The remaining 600 µL was centrifuged again for 10 s, and 300 µL of the resulting supernatant was collected as the cytosolic fraction. The residual supernatant was discarded, and the pellet was resuspended in 1 mL of ice‐cold 0.1% NP‐40 in PBS, centrifuged again for 10 s, and the supernatant was removed. The final pellet (∼20 µL) was designated as the nuclear fraction. Nuclear fractions and whole cell lysates containing DNA were sonicated twice for 5 s each. All samples were stored at −80°C until proteomic analysis (*N* = 4).

### 2.5 Multi‐Enzyme Digestion Filter‐Aided Sample Preparation (MED FASP)

Neuronal cultures (*N* = 5) and hippocampal extracts (*N* = 5) containing 80 µg of total protein were processed using the MED‐FASP (Multi‐Enzyme Digestion Filter‐Aided Sample Preparation) method, as originally described [11] with modifications according to [12]. Proteins were first digested overnight with endoproteinase LysC, followed by a 3‐h digestion with trypsin at 37°C. The enzyme‐to‐protein ratio was 1:40. All digestions were performed in 50 mM Tris‐HCl buffer containing 1 mM DTT, pH 8.5. Aliquots containing 8 µg of total peptide were desalted using C18 Empore Disk membranes (3 M, Saint Paul, MN, USA) following the StageTips (Stop‐and‐Go Extraction Tips) protocol [13]. Final peptide eluates were concentrated to a volume of approximately 5 µL and stored at −20°C until mass spectrometric analysis.

To assess GFAP levels in nuclear and cytoplasmic compartments of neurons, the protocol was modified as follows: protein digestion was carried out overnight with trypsin only (1:40 enzyme‐to‐protein ratio) at 37°C. Peptides were desalted using Pierce C18 Spin Columns (Thermo Fisher Scientific, Waltham, MA, USA), following the same StageTips‐based procedure.

### 2.6 Liquid Chromatography—Tandem Mass Spectrometry

Analysis of peptide mixtures was performed using a QExactive HF mass spectrometer (Thermo‐Fisher Scientific, Palo Alto, CA, USA). Samples containing 1 µg of total peptide were separated on a 50 cm column with 75 µm inner diameter packed C18 material (100 Å pore size; Dr. Maisch GmbH, Ammerbuch‐Entringen, Germany). The peptides were separated using the two‐step acetonitrile gradient: 5%–40% over the first 85 min (300 nL/min) and 40%–95% for the following 15 min (300 nL/min). The temperature of the column was 55°C. The mass spectrometer was operated in data‐dependent mode with survey scans acquired at the resolution of 60,000 at m/z 200 (transient time 256 ms). Up to the top 15 most abundant isotope patterns with a charge ≥+2 from the survey scan (300–1650 m/z) were selected with an isolation window of 1.6 m/z and fragmented by HCD with normalized collision energies of 25. The maximum ion injection times for the survey scan and the MS/MS scans were 20 and 60 ms, respectively. The ion target value for MS1 and MS2 scan modes was set to 3 × 10^6^ and 10^5^, respectively. The dynamic exclusion was 25 s and 10 ppm. The mass spectrometry data has been deposited in the ProteomeXchange Consortium via the PRIDE partner repository [14] with the dataset identifier: PXD025978 (https://proteomecentral.proteomexchange.org/cgi/GetDataset?ID = PXD025978) and PXD038676 (https://www.ebi.ac.uk/pride/archive/projects/PXD038676).

For the assessment of GFAP presence in subcellular fractions of neurons, the procedure was as follows. Samples were spiked with Hi3 *E. coli* and Phos B standards (50 fmol per injection) for label‐free quantification purposes. The resulting peptide mixtures were analyzed using an Acquity I‐Class ultra‐high‐performance liquid chromatography (UHPLC) system, coupled with a Synapt XS ion mobility quadrupole time‐of‐flight mass spectrometer (QTOF‐MS) from Waters. Data were acquired in data‐independent acquisition (DIA) mode using the MSe acquisition method. In function one, low collision energy (6 eV) was used, while in function two, high collision energy with a ramp from 10 to 35 eV was applied for fragmentation. Data acquisition was carried out using an electrospray ionization (ESI) source operated in positive mode, with an acquisition time of 0.4 s per function over the m/z range of 50–2000. Source parameters were as follows: nebulizing and drying gas (nitrogen) flow rates of 650 L/h and 45 L/h, respectively; spray voltage: 3.0 kV; source temperature: 120°C; and desolvation temperature: 350°C. Peptides were separated using a reversed‐phase (C18) Waters HSS T3 chromatographic column (1.0 × 100 mm, 1.8 µm) with a gradient elution of solvent A (water with 0.1% formic acid) and solvent B (acetonitrile with 0.1% formic acid). The total run time was over 180 min at a flow rate of 40 µL/min. The mass spectrometry data has been deposited in the ProteomeXchange Consortium via the PRIDE with the dataset identifier PXD067465 (https://proteomecentral.proteomexchange.org/ui?pxid = PXD067465; for the review: username: reviewer_pxd067465@ebi.ac.uk password: kgHGrvwQzgCt).

### 2.7 Proteomic Data Analysis

The MS data was analyzed using MaxQuant [15] v1.2.6.20. Proteins were identified by searching MS and MS/MS data of peptides against the UniProtKB/Swiss‐Prot database. Carbamidomethylation was set as the fixed modification. The maximum false peptide and protein discovery rate was specified as 0.01. Protein abundances were calculated using the TPA method [4, 16]). In the case of GFAP assessment in subcellular fractions of neurons MS data were analyzed using ProteinLynx Global Server v. 3.03 against the UniProt database (Mouse reviewed proteome, entry date: May 15, 2025) for protein identification and quantification. The following settings were used for data analysis: enzyme specificity—trypsin with a maximum of two missed cleavages; fixed modification—carbamidomethylation; variable modification—methionine oxidation; false discovery rate (FDR) – 4%. Protein abundances were calculated using the TPA method as mentioned above.

### 2.8 Statistical Analysis

All results are presented as mean ± SEM unless we stated otherwise. The statistical analysis was performed using Student's t‐test preceded by Fisher F‐test. We used nonpaired Student's t‐test for comparisons between any two experimental groups. The statistical analyses were performed using SigmaPlot 11 software (Systat Software). Data are expressed as mean ± SEM with significance levels of *p ≤ 0.05, **p ≤ 0.01, ***p ≤ 0.001.

### 2.9 Chemical Induction of Long‐Term Potentiation (cLTP) and Long‐Term Depression (cLTD)

All the experiments were performed on the 14th day of the culture. Prior to the cLTP or cLTD induction, the cultures were transferred to the 37°C Ringer's solution (137 mM NaCl, 5 mM KCl, 1.89 mM CaCl_2_, 10 mM HEPES, 2.5 mM glucose; pH 7.3). Chemical short LTP was induced with addition of 1 µM strychnine and 200 µM glycine, within 10 s incubation [17]. Chemical NMDAR‐dependent LTD was induced using Ringer's solution supplemented with 50 µM N‐methyl‐D‐aspartic acid, within 20 min incubation (NMDA; Sigma‐Aldrich, Saint Louis, USA; M3262), as described in [18, 19, 20]. After chemical stimulation cultures were fixed with 4% PFA in PBS, 15 min, RT.

### 2.10 Immunofluorescence Staining and Confocal Microscopy

Cells were fixed in ice‐cold 4% paraformaldehyde for 15 min and processed for immunofluorescence staining as previously described (Mamczur et al., 2015), with a modification in the permeabilization step. Two protocols were applied, differing in the detergent incubation time: a short protocol (5‐min incubation, Figures 2 and S1) and a long protocol (45‐min incubation, Figures 6, 7 and S1,S2), both using 0.1% Triton X‐100 and 1% BSA in PBS. Subsequently, cells were incubated overnight at 4°C with the following primary antibodies: mouse anti‐CAMK2 pT286 (1:1000; Abcam, Cambridge, UK; ab171095), rabbit anti‐active caspase‐3 (1:800; Sigma‐Aldrich, Saint Louis, USA; C848), chicken anti‐MAP2 (1:1000; Abcam; ab5392), rabbit anti‐GFAP (1:1000; Sigma‐Aldrich; G9269), and mouse anti‐NeuN (1:1000; Abcam; ab104224). Primary antibodies were detected using the following fluorophore‐conjugated secondary antibodies: anti‐rabbit FITC (1:250; Sigma‐Aldrich; F6005), anti‐rabbit Alexa Fluor 488 (1:2000; Invitrogen, USA; A11034) or anti‐rabbit Alexa Fluor 633 (1:2000, Invitrogen, USA; A21071; to detect the GFAP in copGFP control Lentivirus transduced cells—Figure S2A,S2C), anti‐mouse Alexa Fluor 633 (1:2000; Invitrogen; A21046), and anti‐chicken Alexa Fluor 568 (1:2000; Abcam; ab175477). Nuclei were counterstained with Fluoroshield containing DAPI (F6057, Merck, KGaA, Darmstadt, Germany). The copGFP (green fluorescent protein from copepod *Pontellina* sp.) signal was detected directly without immunolabeling. Stained cells were imaged using an Olympus FV1000 confocal microscope (Olympus, Tokyo, Japan) equipped with a Plan Apo 60×/1.4 NA oil‐immersion objective or a 10× dry objective. For each sample, at least 8 images were acquired from randomly selected fields, avoiding slide edges, using the Sequential Scan mode (average nuclei count per field of view was: 220, 50, and 10 for 10x, 20x, and 60x magnifications, respectively).

### 2.11 Western Blot

For Western blot analysis, cell homogenates were prepared from ∼260,000 cells per sample from neuronal cultures (N), astrocyte cultures (A), and astrocyte–neuron co‐cultures (A+N). At day 7 or 14 in vitro (as indicated), cells were washed with PBS and lysed in 1× Laemmli buffer (0.05 M Tris–HCl, pH 6.8, 2% SDS, 10% glycerol, 0.1 M DTT; without bromophenol blue). Lysates were clarified by centrifugation (20,000 × g, 20 min, 4°C), and the supernatants were collected. Total protein concentration was determined using the Bradford assay; final concentrations ranged from 0.6 to 0.7 µg/µl. Aliquots containing 14 µg of total protein were mixed with loading buffer, denatured for 5 min at 99°C, and briefly centrifuged. Proteins were separated by 10% SDS–PAGE and transferred to nitrocellulose membranes using wet transfer.

Membranes were blocked for 1 h in 5% BSA in PBS and incubated overnight at 4°C with primary antibodies diluted in PBS (mouse anti‐GFAP, SAB5201104, Sigma, 1:1,000; rabbit anti‐actin, A2066, Merck, 1:10,000). Membranes were washed with 0.1% Triton X‐100 in PBS (2 × 5 min) followed by PBS (1 × 5 min), and then incubated for 1 h at room temperature with HRP‐conjugated secondary antibodies diluted in PBS (goat anti‐mouse IgG–peroxidase, A4416, Sigma, 1:20,000; goat anti‐rabbit IgG–peroxidase, A6154, Sigma, 1:20,000). Bands were developed using 3,3′‐diaminobenzidine (DAB; 0.05% DAB in 0.02% H_2_O_2_).

The densitometric analysis of protein bands was performed using the ImageJ/Fiji [21] software. The signal from GFAP was normalized to the signal obtained for γ‐actin.

### 2.12 IF Image Analysis

ImageJ/Fiji [21] was used for fluorescence quantification in cultured cells. Cell boundaries were manually delineated, and fluorescence intensity was measured within regions of interest (ROIs) and normalized to ROI area. For immunofluorescence analyses, MAP2 and NeuN were used as neuronal markers to define the total neuronal area and neuron‐specific nuclei, respectively. Neuronal culture purity was determined by calculating the proportion of nuclei double‐positive for NeuN and MAP2 relative to all nuclei in the field of view. For the remaining experiments, MAP2 and DAPI signals were used to define cytoplasmic and nuclear ROIs, respectively, on an image‐by‐image basis. These ROIs were then used to quantify area‐normalized mean fluorescence intensity for pT286 CaMK2, NeuN, caspase‐3, and GFAP in the corresponding channels. For each immunofluorescence experiment, at least three independent biological replicates were performed, with a minimum of eight images acquired per replicate. Depending on magnification, each field of view contained approximately 160–280 cells at 10× (scale bar, 100 µm), 40–60 cells at 20× (scale bar, 50 µm), and up to 10 cells at 60× (scale bar, 20 µm). The experiment‐specific number of analyzed cells (*n*) is provided in the corresponding figure legends.

### 2.13 Measurement of Extracellular Glucose During Hippocampal Neuron Culture

Glucose levels in culture media were measured using a modified protocol based on the Glucose‐Glo Assay Kit (J6021, Promega, USA). Reagents were thawed on ice (Luciferin Detection Solution at room temperature) and mixed gently. Culture medium (25 µL), collected on day 0, 7, 10, and 14 of hippocampal neuron culture, was diluted in PBS (1:100 for 2.5 mM glucose), and mixed with 25 µL of Glucose Detection Reagent. After a 60‐min incubation at room temperature, bioluminescence was measured (n = 3) and corelated to the control medium.

### 2.14 GFAP Silencing

To reduce GFAP protein level, GFAP shRNA (m) lentiviral particles specifically designed to silence the mRNA in mouse cells (sc‐35466, Santa Cruz, USA) was used. The transduction efficiency was assessed with the recommended control virus, copGFP Control Lentiviral Particles (sc‐108084, Santa Cruz, USA), following the manufacturer's instructions. The fluorescence detection of the copGFP protein was the primary indicator of transduction efficiency. The concentrations of viral particles applied resulted in positive detection of copGFP protein in all cells after 48 h post transduction (Figure S2c). The control copGFP lentivirus did not affect cell outgrowth, cell number, or cell morphology when evaluated on days 7 and 14 of neuronal monoculture (Figure S2a,S2c).

Finally, the incubation time with lentiviral particles carrying GFAP shRNA was extended to 72 h to ensure effective silencing of GFAP expression. Neuronal cultures and astrocyte–neuron co‐cultures were transduced on days 4, 7, and 10 for 72 h, enabling assessment of the effects of GFAP gene knockdown on days 7, 10, and 13, respectively (Figures 7a–c and S2a,b). For samples subjected to a 7‐d incubation with GFAP shRNA lentiviral particles, the infection period spanned days 7 to 14 of cell culture (Figures 7d and S2b).

## 3 Results

### 3.1 Proteomic Profiling of Cultured Neurons and Hippocampal Tissue

**FIGURE 1:**
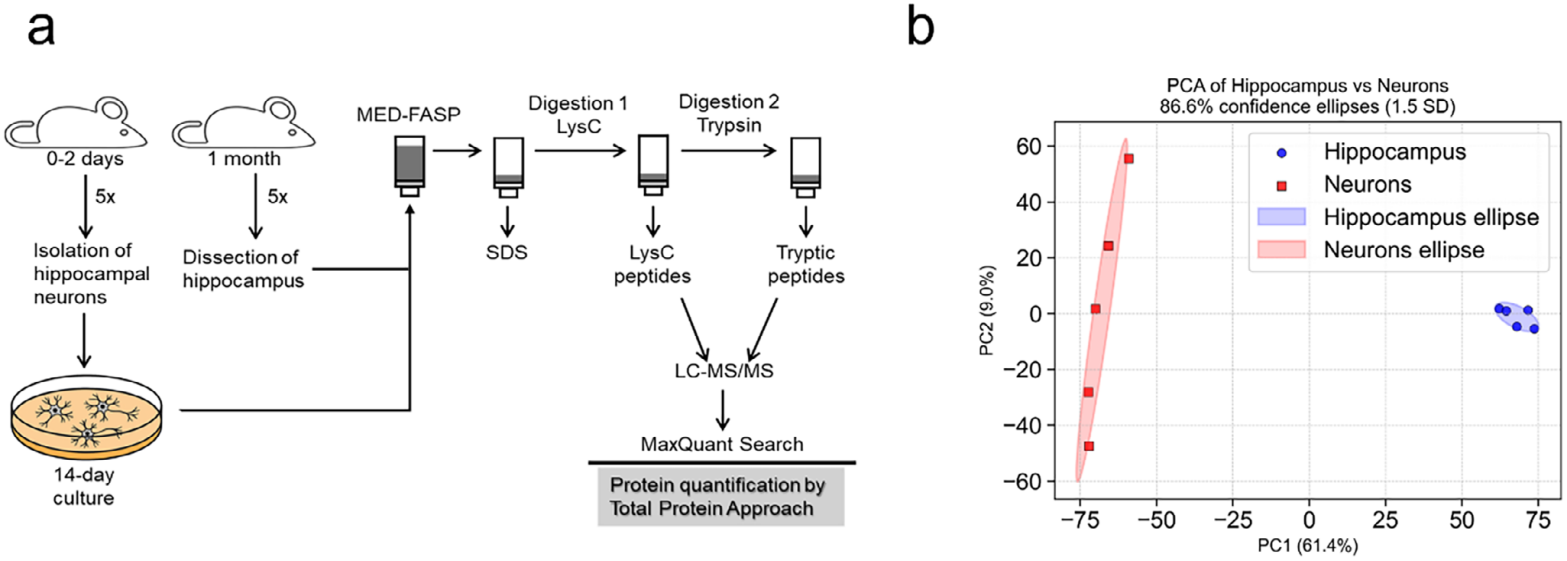
Proteomic analysis of the hippocampus dissected from 1‐month‐old mice and the 14‐day‐old mouse hippocampal neuron cultures. (a) Proteomic workflow. Brain tissue was isolated from 5 animals per group. Hippocampal neurons were isolated from 0–2‐day‐old C57BL6 mice and cultured for 14 days (*N* = 5 biological replicates). The tissue and cell lysates were processed by the MED‐FASP procedure with two steps of enzymatic digestion. Peptides were analyzed by LC‐MS/MS. The spectra were searched using MaxQuant software. Protein abundances were calculated using the “total protein approach” (TPA) method. (b) Principal Component Analysis (PCA) plot of hippocampus and neuron samples based on proteomics data (Supporting Information Table S1). Points represent individual samples; ellipses show 1.5 SD (≈86.6%) confidence intervals. PCA was computed using the whole set of proteins quantified in hippocampal tissue and neuronal cultures (Supporting Information Table S1).

The extracts from 14‐day‐old cultures of hippocampal neurons and hippocampi dissected from 1‐month‐old mice (Figure 1a) were analyzed by global proteomics. The label‐free TPA method was employed to focus on similarities and differences in proteomes of hole hippocampi and primary cultures of hippocampal neurons. This method enables absolute quantification of protein concentrations without the use of labeling, based on the summed MS signal of all peptides normalized to the total protein amount in each sample [22].

We quantified 7,294 proteins in hippocampal tissue and 5,537 proteins in hippocampal neuronal cultures (criterion: ≥1 unique peptide) (Table S1). The overlap between the two datasets comprises 5,536 proteins quantified in both sample types. 1,758 proteins were detected only in hippocampal tissue, whereas 1 protein was detected only in neuronal cultures. For the analyses we retained proteins quantified in at least 3 biological replicates in at least one group. For between‐group statistical testing, proteins were tested only when quantified in at least 3 biological replicates in each compared group; otherwise, they were excluded from statistical inference.

For Figure 1b, the PCA was performed using whole set of proteins quantified in both datasets, based on the protein concentrations derived from TPA values.

Importantly, as expected, a principal component analysis of protein concentrations revealed substantial differences between the hippocampus and cultured hippocampal neurons (Figure 1b).

### 3.2 Purity and Functionality of Hippocampal Neuronal Cultures

**FIGURE 2:**
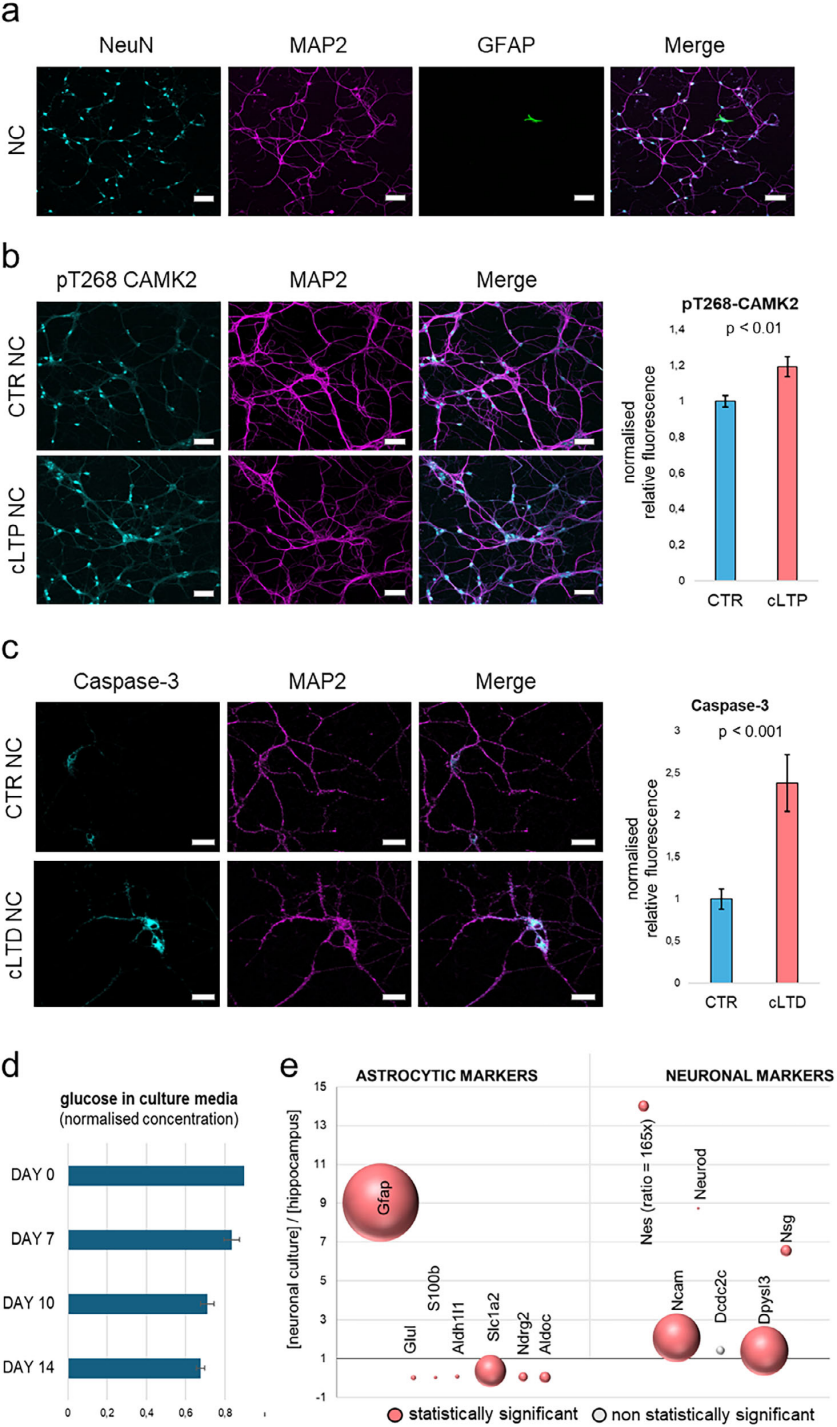
Purity and functionality of hippocampal neurons. (a) Exemplary confocal images of pure neuronal culture (NC) monitored by expression of neuronal (NeuN and MAP2) and astrocytic (GFAP) markers. The cells were permeabilized with Triton X‐100 for 5 min. Scale bar = 50 µm. (b) Neurons cultured in near‐physiological glucose concentration (2.5 mM) are susceptible to the induction of chemical long‐term potentiation (cLTP). Representative confocal images show phospho‐CAMK2 (Thr286) as a marker of the early phase of cLTP, and MAP2 as a neuronal marker. The chart presents quantification of the pCAMK2 phosphorylation changes. Scale bar = 50 µm. (c) Neurons cultured in 2.5 mM glucose are susceptible to the induction of chemical long‐term depression (cLTD). Representative confocal images show cleaved Caspase‐3 as a marker of cLTD, and actin as a structural marker of neuronal morphology. The chart presents quantification of Caspase‐3 activation (*N* = 3 (biological replicates), *n* = 140 (number of analyzed cells)). Scale bar = 20 µm. (d) Changes in glucose level in the culture medium during the growth of pure neuronal cultures (*N* = 3). (e) Differences in the concentration of canonical astrocytic and neuronal molecular markers between hippocampal neuron cultures and whole hippocampal tissue extracts (*N* = 5). Bubble plots display the ratios of protein concentrations in neuronal cultures relative to whole hippocampus. Bubble size is proportional to the average protein concentration. Red bubbles represent proteins whose concentrations differ significantly between neuronal cultures and hippocampal tissue. Detailed data are provided in Supporting Information Table S1.

To minimize the presence of glial cells in primary hippocampal cultures derived from neonatal mouse hippocampi, 10 µM cytosine β‐D‐arabinofuranoside (AraC) was added to the culture medium and maintained throughout the entire culture period. Immunofluorescence analysis revealed that nonneuronal cells accounted for less than 5% of the total cell population (see representative image in Figure 2a).

To assess whether neurons cultured in near‐physiological, 2.5 mM glucose exhibit physiological properties typical of neuronal cultures—such as the capacity to express basic forms of cellular and molecular plasticity—we examined the effects of chemically induced cLTP and cLTD on established plasticity markers: phosphorylation of calcium/calmodulin‐dependent protein kinase II (CAMK2) at threonine residue 286 and activation of caspase‐3, respectively (see references: [23, 24]). We observed that the induction of cLTP and cLTD significantly increased fluorescence signals corresponding to these markers (Figure 2b,c) demonstrating that neurons cultured in 2.5 mM glucose retain key molecular signatures of activity‐dependent plasticity, thus validating the physiological relevance of this in vitro model. Notably, culturing neurons in 2.5 mM glucose for 14 days resulted in only ∼30% reduction in glucose concentration in the culture medium, indicating that the glucose supply remained sufficient throughout the experimental period (Figure 2d).

### 3.3 Neuronal and Astrocytic Markers in Cultured Neurons and Intact Hippocampi

Mass spectrometry‐based proteomic analysis revealed that the concentrations of several neuronal markers—including nestin, neurogenic differentiation factors 1 and 2 (NeuroD1, NeuroD2), neural cell adhesion molecules 1 and 2 (NCAM1, NCAM2), dihydropyrimidinase‐related protein 3 (DPYSL3), and neuron‐specific protein family member 1 (NEFM1)—were significantly higher in 14‐day‐old hippocampal neuronal cultures compared to whole hippocampi of young mice (Figure 2e and Table S1). These findings are consistent with the heterogeneous cellular composition of the hippocampus, where neurons account for approximately 50% of the total cell population [25].

In contrast, analysis of canonical astrocytic markers showed their substantially lower concentrations in the neuronal cultures compared to whole hippocampi [26]. Specifically, glutamine synthetase (GLUL/GS) was present at approximately 1% of its hippocampal level, S100β at ∼2%, NDRG2 at ∼5%, cytosolic 10‐formyltetrahydrofolate dehydrogenase (ALDH1L1) at ∼10%, and excitatory amino acid transporter 2 (EAAT2/GLT‐1) at ∼30% (Figure 2e and Table S1). Our study also unequivocally confirmed the hypothesis that so called “brain isozyme of aldolase”—aldolase C (ALDOC) is expressed almost exclusively by nonneuronal cells of hippocampus and can be considered a marker of astrocytes (Figure 2e and Table S1 and [26, 27].

Unexpectedly, the concentration of GFAP in nominally pure neuronal cultures was approximately 10‐fold higher than that measured in whole hippocampi (Figure 2e and Table S1). Notably, although GFAP levels in hippocampal tissue were consistent with those reported in the literature [28] the abundance of GFAP in the cultured neurons was similar to the abundance of the main metabolic enzymes. This unexpected observation could raise concerns about potential contamination or measurement artifacts. However, GFAP was confidently quantified in neuronal cultures based on over 40 GFAP‐specific peptides, including three unique peptides not shared with any other protein. To validate the result originally obtained at the Max Planck Institute of Biochemistry in Martinsried (Germany), we conducted additional measurements in an independent laboratory at the Omics Research Center of Wroclaw Medical University (Poland). The analysis confirmed that the total GFAP concentration in pure hippocampal neuron cultures was several fold (approximately fivefold) higher than that typically observed in whole hippocampal tissue (Figure 6a, and Table S1).

Given the unexpected magnitude of GFAP expression, we performed a more detailed analysis of GFAP abundance in hippocampal neurons, the results of which are presented in a dedicated section 3.6. “Insight into the role of GFAP in neurons”.

### 3.4 Glucose and Energy Metabolism

#### 3.4.1 Expression of Glucose Transporters and Glycolytic Enzymes, and Lactate Metabolism in Cultured Neurons Compared to Hippocampal Tissue Samples

**FIGURE 3:**
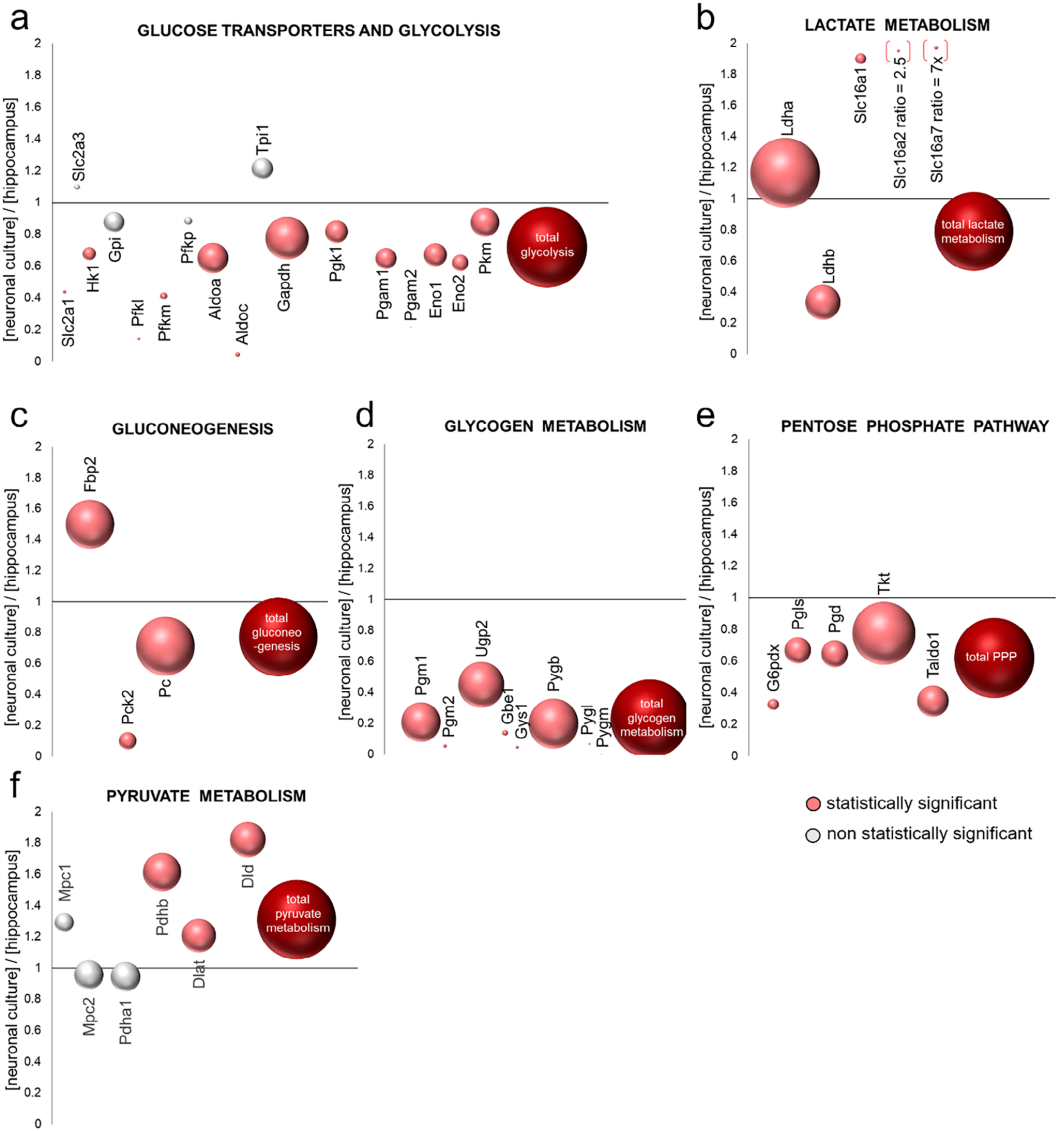
Differences in the concentration of glucose and glycogen metabolism proteins between hippocampal neuron cultures and whole hippocampal tissue extracts. (a) Glucose transporters and glycolysis. (b) Lactate metabolism. The changes for the proteins Slc16a2 and Slc16a7, which exceed the presented scale, are reported separately. (c) Gluconeogenesis. (d) Glycogen metabolism. (e) Pentose Phosphate Pathway. (f) Pyruvate metabolism. Bubble plots display the ratios of protein concentrations in neuronal cultures relative to whole hippocampus. Bubble size is proportional to the average protein concentration. Red bubbles represent proteins whose concentrations differ significantly between neuronal cultures and hippocampal tissue. Detailed data are provided in Supporting Information Table S2.

Our quantitative proteomic analysis revealed that the concentrations of glucose transporters and glycolytic enzymes were significantly lower in 14‐day‐old hippocampal neuronal cultures compared to whole hippocampal tissue (Figure 3a and Table S2). This observation aligns with the established understanding that glial cells, particularly astrocytes, exhibit a more glycolytic phenotype than neurons. Astrocytes predominantly express glucose transporter 1 (GLUT1) and possess high levels of glycolytic enzymes, facilitating efficient glucose uptake and metabolism. In contrast, neurons primarily express glucose transporter 3 (GLUT3) and rely more on oxidative phosphorylation, with a comparatively lower glycolytic capacity.

Furthermore, the aggregate concentrations of glycolytic enzymes in the hippocampal tissue were comparable to those reported in previous studies employing the TPA, such as the work by [28] This consistency underscores the high reproducibility and reliability of TPA‐based quantitative proteomics in assessing protein expression levels across different experimental setups.

The analysis of glycolytic enzyme expression in hippocampal neurons revealed several notable and unexpected findings. First, contrary to previous reports suggesting that neurons predominantly express the platelet‐type isoform of phosphofructokinase (PFKP) [29], we observed that in cultured neurons, the expression level of PFKP was comparable to that of the muscle isoform, PFKM (Figure 3a and Table S2). This finding challenges the notion of strict isoform specificity and suggests a more heterogeneous expression pattern. Interestingly, the concentration of PFKP in hippocampal tissue was nearly identical to that observed in pure neuronal cultures, indicating that PFKP expression in the hippocampus is likely confined to neuronal cells. In contrast, PFKM was the dominant isoform in hippocampal tissue, with PFKP and the liver isoform, PFKL, present at substantially lower but comparable levels. Only trace amounts of PFKL were detected in cultured neurons, suggesting minimal contribution from nonneuronal cell types.

Phosphofructokinase‐1 (PFK1) is a key regulatory enzyme of glycolysis, catalyzing the irreversible phosphorylation of fructose‐6‐phosphate to fructose‐1,6‐bisphosphate. The distribution of its isoforms—PFKP, PFKM, and PFKL—reflects the distinct energetic and metabolic requirements of different cell types. Notably, PFKM is adapted to support high glycolytic flux: it exhibits the highest maximal catalytic rate (Vmax) and substrate affinity among the isoforms, and it is the least sensitive to feedback inhibition by ATP or modulation by allosteric effectors [30]. Thus, the elevated expression of PFKM in the hippocampal tissue compared to cultured neurons likely reflects the contribution of astrocytes and other glycolytically active glial cells.

In hippocampal tissue from young mice, the concentrations of lactate dehydrogenase (LDH) isozymes LDHA and LDHB were comparable (Figure 3b and Table S2), aligning with previous proteomic studies [7, 28]. The astrocyte‐neuron lactate shuttle (ANLS) hypothesis posits that astrocytes predominantly express LDHA to convert glycolysis‐derived pyruvate to lactate, which is then shuttled to neurons. Neurons, in turn, express LDHB to convert lactate back to pyruvate, supporting oxidative metabolism [31]. This metabolic coupling is essential for synaptic plasticity phenomena such as LTP [32, 33].

Contrary to expectations, our analysis revealed that in primary hippocampal neuronal cultures, LDHA was the predominant LDH isoform, suggesting an adaptation to the in vitro environment where neurons may rely more on glycolytic pathways for energy production (Figure 3b and Table S2). This shift could be attributed to the absence of astrocytic support and the specific culture conditions, including glucose availability.

Furthermore, we observed that monocarboxylate transporter 1 (MCT1), encoded by the SLC16A1 gene, was the predominant lactate transporter expressed in these neuronal cultures. In the hippocampus, MCT1 is expressed in astrocytes, oligodendrocytes, and certain neuronal populations, facilitating lactate transport between these cells [34]. The presence of MCT1 in neuronal cultures may reflect another adaptive response to the altered metabolic environment in vitro.

#### 3.4.2 Gluco‐ and Glyconeogenic Enzymes in Hippocampal Tissue and Neuronal Culture

Our proteomic analysis revealed the unexpected presence of enzymes traditionally associated with gluconeogenesis and glyconeogenesis—namely, pyruvate carboxylase (PC), phosphoenolpyruvate carboxykinase (PCK), and fructose 1,6‐bisphosphatase isozyme 2 (FBP2)—in pure hippocampal neuronal cultures (Figure 3c and Table S2). While gluconeogenic pathways have been well‐characterized in astrocytes, their activity in neurons remains underexplored. The presence of these enzymes in neurons may reflect their involvement in alternative metabolic pathways beyond canonical gluconeogenesis. It can be hypothesized that their activity contributes to the generation of glucose‐6‐phosphate, a key substrate for the pentose phosphate pathway, thereby supporting NADPH production and maintaining redox homeostasis in neuronal cells.

In addition, some gluconeogenic enzymes, such as FBP2, have been shown to exert multifunctional, nonenzymatic roles in cells. FBP2, in particular, has been implicated in the induction and maintenance of LTP, a cellular mechanism underlying memory formation. Specifically, FBP2 interacts with neuronal CAMK2, and its modulation affects CAMK2 autophosphorylation, a critical step in LTP formation. Silencing or altering the oligomeric state of FBP2 impairs LTP induction and the expression of late‐phase LTP markers, such as c‐Fos and c‐Jun [35, 36]. From this perspective, the presence of FBP2 in neuronal cultures is not unexpected. What is surprising, however, is the inability to detect this protein in hippocampal tissue samples using MS‐based proteomic analysis, despite its demonstrated functional importance in neurons.

#### 3.4.3 Glycogen Metabolism

The average concentration of glycogen metabolism enzymes in cultured hippocampal neurons was found to be significantly lower than in hippocampal tissue (Figure 3d and Table S2). This observation aligns with the longstanding belief that neurons possess limited capacity for glycogen synthesis, whether from exogenous glucose or noncarbohydrate precursors.

Historically, the physiological role of glycogen metabolism in neurons has been associated primarily with embryonic development and certain neurological disorders, such as Lafora disease—a fatal, autosomal recessive glycogen storage disorder characterized by the accumulation of aberrant glycogen inclusions in neurons [37, 38, 39]. However, emerging evidence indicates that mature neurons do possess active glycogen metabolism. Studies have demonstrated that neurons express the brain isoform of glycogen phosphorylase, enabling them to metabolize glycogen [38]. Notably, neuronal glycogen metabolism has been shown to confer protection against hypoxia‐induced neuronal death, suggesting a role in enhancing neuronal tolerance to hypoxic stress [40]. Therefore, the low but detectable levels of glycogen metabolism enzymes observed in neuronal cultures may reflect an adaptive mechanism that contributes to neuronal resilience under hypoxic conditions.

#### 3.4.4 Pentose Phosphate Pathway Activity in Hippocampal Tissue and Neuronal Culture

The concentrations of all enzymes involved in the pentose phosphate pathway (PPP) were significantly higher in hippocampal tissue compared to cultured hippocampal neurons (Figure 3e and Table S2). This finding supports the hypothesis that astrocytes play a central role in sustaining redox balance in the brain by generating NADPH, which is essential for the synthesis of reduced glutathione (GSH). Although neurons have a limited capacity to produce NADPH, they rely on antioxidant support from astrocytes. Rather than importing intact glutathione, neurons take up GSH breakdown products—mainly cysteine and cysteinylglycine—following extracellular degradation mediated by γ‐glutamyl transpeptidase on astrocytic membranes [41, 42]. These precursors are then used for *de novo* synthesis of neuronal GSH.

Notably, we found that the concentration of glucose‐6‐phosphate dehydrogenase (G6PD), the rate‐limiting enzyme of the oxidative branch of the PPP, was approximately threefold lower in cultured neurons compared to hippocampal tissue. This result is consistent with prior studies reporting that PPP activity is approximately 4–5 times higher in astrocytes than in neurons [43].

#### 3.4.5 Pyruvate Catabolism

We observed that the expression levels of key pyruvate dehydrogenase complex (PDC) subunits—including PDHB, PDHX, DLAT, and DLD—were significantly higher in pure hippocampal neuronal cultures than in hippocampal tissue (Figure 3f and Table S2). This suggests that neurons are the principal cellular source of PDC expression in the hippocampus, consistent with their reliance on oxidative metabolism. In contrast, the lower expression levels in hippocampal tissue likely reflect the inclusion of astrocytes and other nonneuronal cells, which are known to rely more heavily on glycolytic metabolism and lactate export [44, 45].

#### 3.4.6 Tricarboxylic Acid Cycle

**FIGURE 4:**
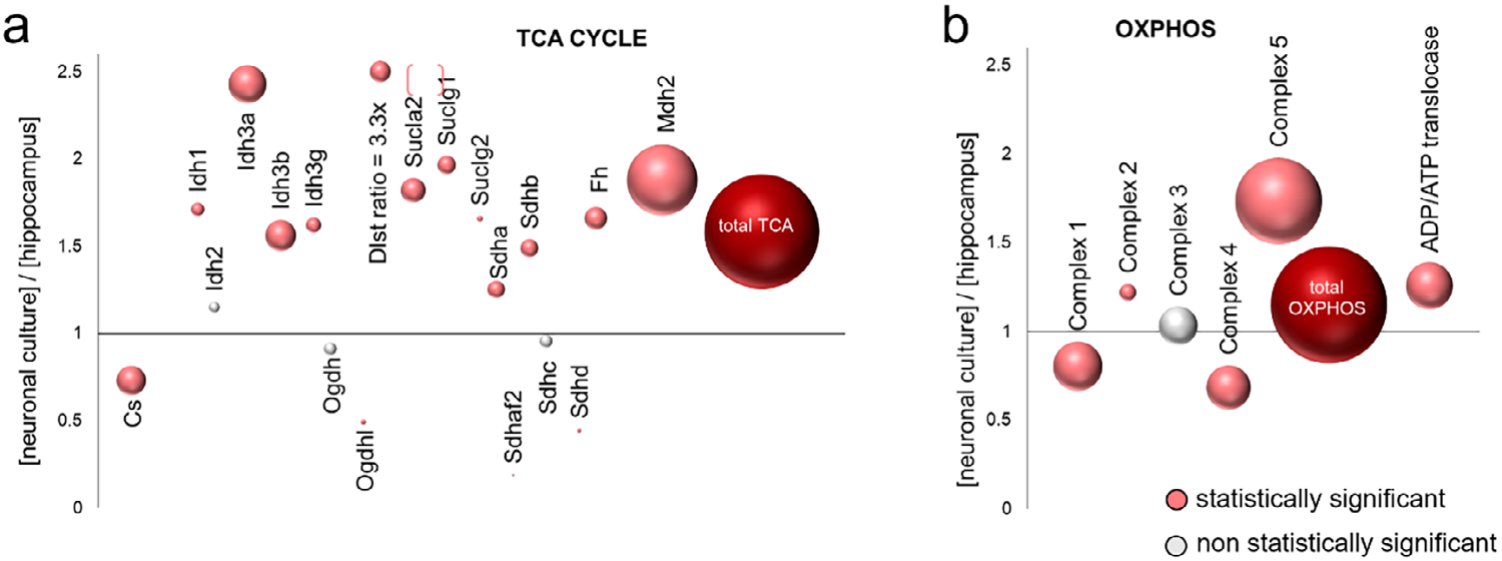
Differences in the concentration of oxidative metabolism proteins between hippocampal neuron cultures and whole hippocampal tissue extracts. (a) Tricarboxylic acid (TCA) cycle. (b) Oxidative phosphorylation (OXPHOS). Bubble plots display the ratios of protein concentrations in neuronal cultures relative to whole hippocampus. Bubble size is proportional to the average protein concentration. Red bubbles represent proteins whose concentrations differ significantly between neuronal cultures and hippocampal tissue. Detailed data are provided in Supporting Information Table S2.

The tricarboxylic acid (TCA) cycle is a sequence of chemical reactions that convert acetyl‐CoA—primarily derived from glycolysis and the β‐oxidation of free fatty acids—into carbon dioxide and water, while generating energy‐carrying molecules such as NADH, FADH_2_, and ATP. Overall, we found that sum of titers of TCA enzymes in pure neuronal culture was significantly, by about 30%, higher than in hippocampus (Figure 4a and Table S2). Among the proteins with the most increased abundance were: isocitrate dehydrogenases (IDH3α and IDH3β), dihydrolipoyllysine‐residue succinyltransferase component of 2‐oxoglutarate dehydrogenase complex (DLST), succinyl‐CoA ligase subunits (SUCLA2, SUCLG1, and SUCLG2), and mitochondrial malate dehydrogenase (MDH2). DH3α and IDH3β, subunits of the NAD^+^‐dependent isocitrate dehydrogenase 3 (IDH3), and DLST catalyze an irreversible step in the TCA cycle, thereby regulating its flux (see in [46]. Their high expression levels in neurons reflect the cells' reliance on oxidative metabolism for ATP production. Succinyl‐CoA ligase does not catalyze a regulatory step in the TCA cycle; however, its high expression ensures efficient progression of the cycle and supports sustained oxidative metabolism (see in [46]. An almost twofold increase in MDH2 abundance in neurons compared to the hippocampus is consistent with changes observed in the total levels of TCA cycle enzymes (Figure 4a and Table S2). This elevation does not correlate with a corresponding increase in cytoplasmic MDH1 expression (Table S2), which would otherwise suggest an upregulation of the malate‐aspartate shuttle—a mechanism that facilitates the transfer of redox equivalents from glycolysis into the mitochondrial TCA cycle.

#### 3.4.7 Oxidative Phosphorylation—OXPHOS

Quantitative comparison of OXPHOS complex proteins revealed a pronounced increase in Complex V (ATP synthase) levels in cultured neurons compared to hippocampal tissue (1125.7 vs. 649.1 pmol/mg, respectively (Figure 4b and Table S2). This increase exceeded the relative changes observed in other complexes (I–IV), whose levels remained similar or were moderately decreased in neurons (Figure 4b and Table S2). Notably, the abundance of ADP/ATP translocases was also higher in cultured neurons compared to hippocampal tissue (330 vs. 260 pmol/mg), indicating an elevated capacity for ATP export from mitochondria. These findings suggest a neuron‐specific adaptation characterized by enhanced oxidative phosphorylation efficiency and ATP production potential. The disproportionate upregulation of Complex V may reflect increased energy demands related to neurotransmission, membrane potential maintenance, and synaptic vesicle recycling, particularly in excitatory glutamatergic hippocampal neurons with high metabolic rates, in the absence of astrocytes, which typically provide metabolic support to neurons.

### 3.5 Proteins Involved in Neuronal Plasticity and Signal Transduction

#### 3.5.1 Glutamatergic Transmission

**FIGURE 5:**
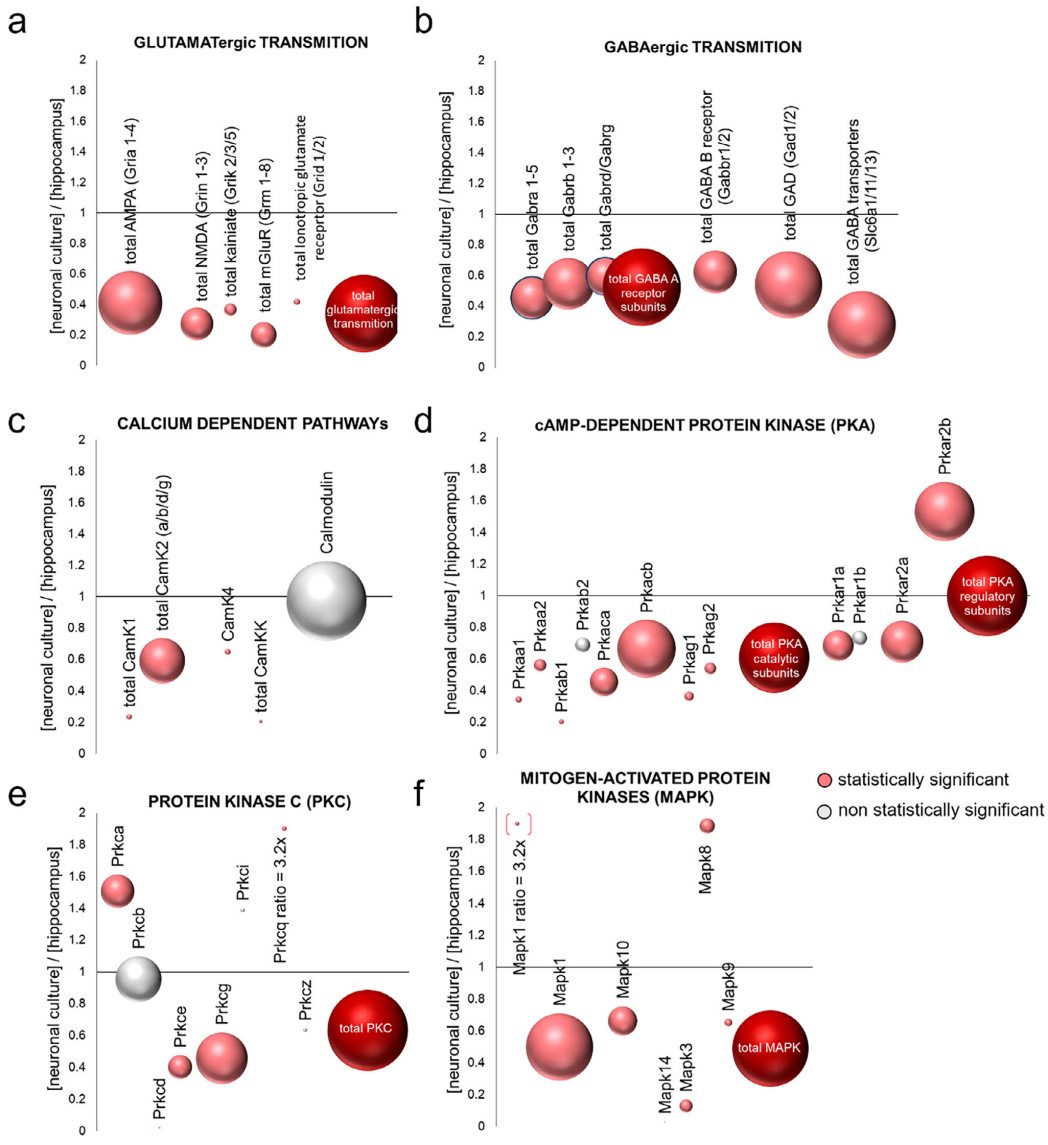
Differences in the concentrations of proteins involved in neuronal plasticity and signal transduction between hippocampal neuron cultures and whole hippocampal tissue extracts. (a) Glutamatergic signaling. (b) GABAergic transmission. (c) Calmodulin and Calcium/Calmodulin‐Dependent Kinases. (d) cAMP‐Dependent Protein Kinase (PKA). (e) Protein Kinase C (PKC). (f) Mitogen‐activated protein kinases (MAPK). Bubble plots display the ratios of protein concentrations in neuronal cultures relative to whole hippocampus. Bubble size is proportional to the average protein concentration. Red bubbles represent proteins whose concentrations differ significantly between neuronal cultures and hippocampal tissue. Detailed data are provided in Supporting Information Table S3.

Glutamate is the main excitatory neurotransmitter in hippocampus, acting through ionotropic and metabotropic receptors. The ionotropic receptors fall into one of four classes: α‐amino‐3‐hydroxy‐5‐methyl‐4‐isoxazolpropionic acid receptors (AMPA receptors, GRIA), *N*‐methyl‐*D*‐aspartate receptors (NMDA receptors, GRIN), kainate receptors (GRIK) and delta receptors (GRID). Metabotropic glutamate receptors (GRM) are represented by several isoforms. Our data revealed significantly lower levels of both receptor types in cultured hippocampal neurons compared to whole hippocampal tissue (Figure 5a and Table S3). This is consistent with previous findings showing that embryonic hippocampal neurons in vitro do not express mature synaptic AMPA and NMDA receptor subunits until several weeks in culture, reflecting their delayed functional maturation [47]. This discrepancy may also reflect the reduced cellular diversity and the absence of glial support in vitro, as well as the lack of complex synaptic architecture and network activity, which are known to influence glutamatergic expression [48].

#### 3.5.2 GABAergic Transmission

γ‐aminobutyric acid (GABA) is the most abundant inhibitory neurotransmitter in the brain, acting through two main types of receptors: the ionotropic GABA_A_ receptor, which functions as a chloride channel, and the metabotropic GABA_B_ receptor. GABA_A_ receptors are pentameric ligand‐gated ion channels composed of various subunits, including α (Gabra), β (GABRB), γ (GABRG), and δ (GABRD) [49]. In contrast, the GABA_B_ receptor is a heterotrimeric G protein‐coupled receptor formed by the GABBR1 and GABBR subunits [50]. Our analysis demonstrated that, similar to glutamatergic transmission, all detected components of the GABAergic system were significantly downregulated in neuronal cultures compared to hippocampal tissue (Figure 5b and Table S3). This reduction extended not only to GABA receptor subunits but also to key enzymes and transporters involved in GABA synthesis and uptake, including glutamate decarboxylases (GAD1 and GAD2) and sodium‐ and chloride‐dependent GABA transporters (SLC6A1, SLC6A11, and SLC6A13).

#### 3.5.3 Calmodulin and Calcium/Calmodulin‐Dependent Kinases

The binding of calcium ions to calmodulin (CALM), followed by the activation of calcium/calmodulin‐dependent protein kinases (CAMKs) and calcium/calmodulin‐dependent protein kinase kinases (CAMKKs), represents the initial stage in the transduction of calcium signaling into various forms of synaptic plasticity (for review, see [51, 52].Our study showed that, with the exception of CALM, the abundances of all detected members of the CAMK family were significantly reduced in neuronal cultures compared to the hippocampal formation (Figure 5c and Table S3). CAMK2 is one of the most abundant proteins across all brain structures [53, 54, 55]. It plays a key role in the early phases of memory formation: glutamate‐induced Ca^2^^+^ influx through NMDA receptors leads to activation of CAMK2 by calcium‐bound calmodulin (a process likely facilitated by FBP2 [35], which in turn promotes the insertion of AMPA receptors into the postsynaptic membrane. [56, 57].

The molecular role of CAMKKs and CAMK4 in synaptic plasticity is different than CAMK2. CAMKKs phosphorylate and regulate the activity of CAMK proteins while active CAMK4 localizes in the cell nucleus and regulates the transcription of genes involved in the late phase of memory formation [52]. The observed downregulation of CAMK and CAMKK proteins in neuronal cultures—despite preserved levels of calmodulin—may reflect impaired intracellular propagation of calcium signals, potentially limiting the capacity of cultured neurons to undergo synaptic plasticity. This is consistent with the immature functional phenotype of in vitro neurons and may be further exacerbated by the absence of coordinated network activity and glial support. Given the critical role of CAMKs in memory‐related processes and AMPA receptor trafficking, these findings suggest that hippocampal neuron cultures may not fully recapitulate the calcium‐dependent plasticity mechanisms observed in intact brain tissue.

#### 3.5.4 cAMP‐Dependent Protein Kinase

cAMP‐dependent protein kinase (PKA, also known as PRKA) is a highly conserved serine/threonine kinase with broad tissue distribution and relatively low substrate specificity. It is capable of phosphorylating various subunits of AMPA and NMDA receptors, thereby modulating their function [58]. In our analysis, we observed that the concentrations of all PKA catalytic subunits were significantly lower in neuronal cultures compared to hippocampal tissue (Figure 5d and Table S3). In contrast to the catalytic subunits, the concentration of the regulatory subunit of PKA did not differ significantly between neuronal cultures and hippocampal tissue.

#### 3.5.5 Protein Kinase C

PKC comprises a family of serine/threonine kinases activated by diacylglycerol (DAG), Ca^2^^+^, and phospholipids. Among the conventional PKC isoforms, PKCα, PKCβ, and PKCγ are Ca^2^^+^‐dependent and play pivotal roles in synaptic plasticity, particularly during the early phases of LTP [59, 60]. Our analysis revealed that PKCα and PKCβ were abundantly expressed in both cultured neurons and hippocampal tissue, with PKCγ expression being notably higher in hippocampal tissue. Notably, while the concentration of PKCα was slightly elevated in cultured neurons, the combined levels of Ca^2^^+^‐dependent PKC isoforms were nearly twofold higher in hippocampal tissue compared to neuronal cultures (Figure 5e and Table S3). This differential expression underscores the significance of PKC isoforms in hippocampal synaptic function and plasticity [61].

#### 3.5.6 Mitogen‐Activated Protein Kinases

Mitogen‐activated protein kinase (MAPK) signaling is essential for long‐term potentiation, particularly in translating transient electrical stimuli into lasting structural and transcriptional modifications. Among the MAPK family, MAPK1 and MAPK3 (also known as ERK2 and ERK1, respectively) are the primary isoforms implicated in hippocampal LTP, where they regulate gene expression, protein synthesis, and long‐term memory formation [62]. Our analysis revealed that the concentrations of these kinases were more than twofold higher in hippocampal tissue than in cultured neurons (Figure 5f and Table S3), suggesting that neurons maintained in vitro may exhibit an immature molecular phenotype with limited capacity for activity‐dependent plasticity.

### 3.6 Insight Into the Role of GFAP in Neurons

**FIGURE 6:**
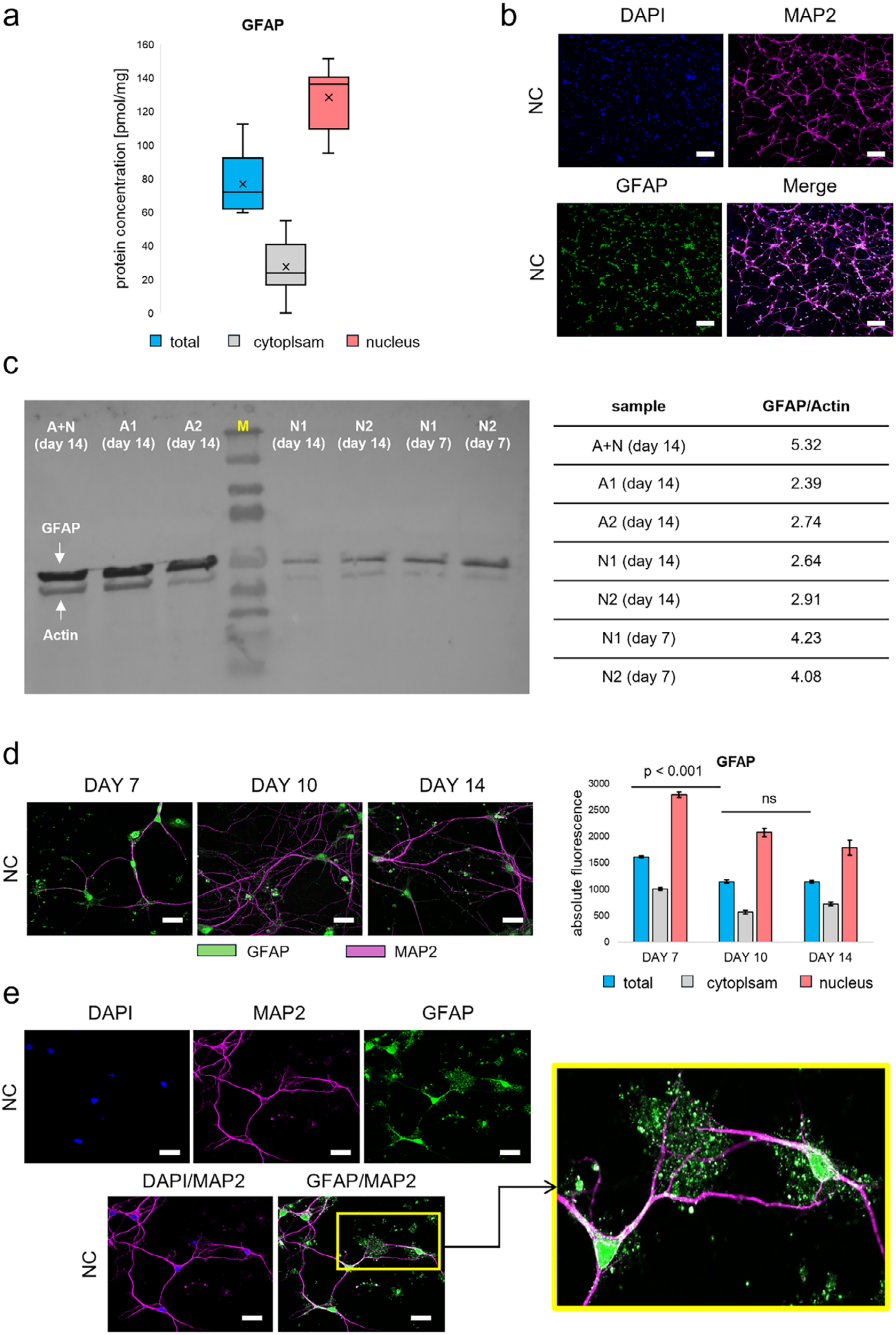
Insight into the role of GFAP in neurons. (a) Mass spectrometry analysis of GFAP concentration in neuronal culture (NC; 14‐day old). The chart shows GFAP titer in the extract from the whole neurons and form nuclear and cytoplasmic fractions (*N* = 4). (b) MAP2‐ and GFAP‐related immunofluorescent signals observed in neurons subjected to prolonged permeabilization with Triton X‐100 (45 minutes) to expose the maximal number of epitopes. GFAP‐associated fluorescence is restricted to the MAP2‐positive region in the merged channel. Scale bar = 100 µm. (c) Western blot analysis of GFAP protein in extracts from: a 14‐day astrocyte–neuron co‐culture (A+N day 14), two 14‐day astrocyte monocultures (A1 day 14, A2 day 14), and neuronal cultures (7‐day: N1 day 7, N2 day 7; and 14‐day: N1 day 14, N2 day 14). The table shows the ratio of GFAP to actin in the analyzed samples. (d) Changes in GFAP‐associated fluorescence signal during the course of neuronal culture (NC). The chart shows GFAP‐related fluorescence in neurons, including their cytoplasm and nuclei, measured on days 7, 10, and 14 of culture. GFAP fluorescence was not normalized to MAP2‐related signal, as MAP2 expression changes significantly over time due to neurite outgrowth and network development (*N* = 3, *n* = 190 cells per culture day point). Scale bar = 20 µm. (e) Outside the nuclei, the GFAP‐related signal was particularly pronounced around newly forming intercellular connections. Scale bar = 20 µm.

MS‐based proteomic analysis revealed that GFAP concentration was markedly higher in cultured hippocampal neurons than in hippocampal tissue (Figure 2e and Table S1). To validate this finding, we repeated the MS‐based measurements in an independent laboratory using a newly prepared hippocampal neuron cultures. The analysis confirmed the high concentration of GFAP in these cells (Figure 6a and Table S1). GFAP concentration was especially elevated in the neuronal nuclei, reaching levels more than 4 times higher than those observed in the cytoplasm of hippocampal neurons (Figure 6a).

To further investigate this unexpected finding, we compared GFAP levels in pure neuronal cultures, astrocyte monocultures, and astrocyte‐neuron co‐cultures using immunofluorescence (IF) and Western blotting (WB). As demonstrated in Figure 2a, the standard IF sample preparation protocol—fixation for 15 min in 4% paraformaldehyde (PFA) followed by 5‐min permeabilization with 0.1% Triton X‐100 in the presence of 1% BSA—revealed GFAP localization exclusively in astrocytes. The apparent absence of GFAP in neurons was in stark contrast to the results obtained using MS‐based quantification (Figures 2e,6a and Table S1).

To address this discrepancy, we employed an extended Triton X‐100 permeabilization step (45 min), a technique that may expose epitopes not accessible during the standard 5‐min permeabilization of fixed cells [63]. IF staining of samples prepared with this modified protocol validated the MS‐based measurements demonstrating that GFAP was abundantly expressed in neurons cultured in the absence of astrocytes (Figure 6b). In line with the above results, WB analysis confirmed the presence of GFAP in pure neuronal cultures (Figure 6c). Quantitative analysis of the WB revealed that, relative to γ‐actin, GFAP levels were higher in neurons than in astrocytes on day 7 of culture, and similar on day 14 (Figure 6c). Importantly, proteomic data also showed that γ‐actin levels are approximately 40% higher in cultured neurons compared to hippocampal tissue (Table S1), suggesting that even on day 14, GFAP levels in neurons remain elevated relative to astrocytes. Conversely, GFAP levels in astrocyte‐neuron co‐cultures were higher than in either neuronal or astrocytic monocultures and the presence of this protein was largely restricted to astrocytes (Figure 6c). This observation was consistent with the results of IF staining, which revealed robust GFAP expression in astrocytes co‐cultured with neurons—using both, the 5 and 45 min‐long permeabilization with Triton X‐100 (Figure S1a).

The Western blot analysis also demonstrated decreasing amount of GFAP with increasing neuronal culture time. The ratio of GFAP to γ‐actin was higher on day 7 than on day 14 of culturing (Figure 6c). This observation was consistent with the results of IF experiments, which showed the strongest GFAP‐associated fluorescence signal in neurons on day 7 of culture, and markedly reduced signal on day 14 (Figure 6d). Detailed image analysis confirmed the MS‐based findings, showing that GFAP is predominantly localized in the neuronal soma, including the nuclei (Figure 6d).

Notably, a substantial amount of GFAP was also observed within and around newly forming intercellular connections (Figure 6e), which may indicate a potential role for GFAP in the formation of neuronal networks.

**FIGURE 7:**
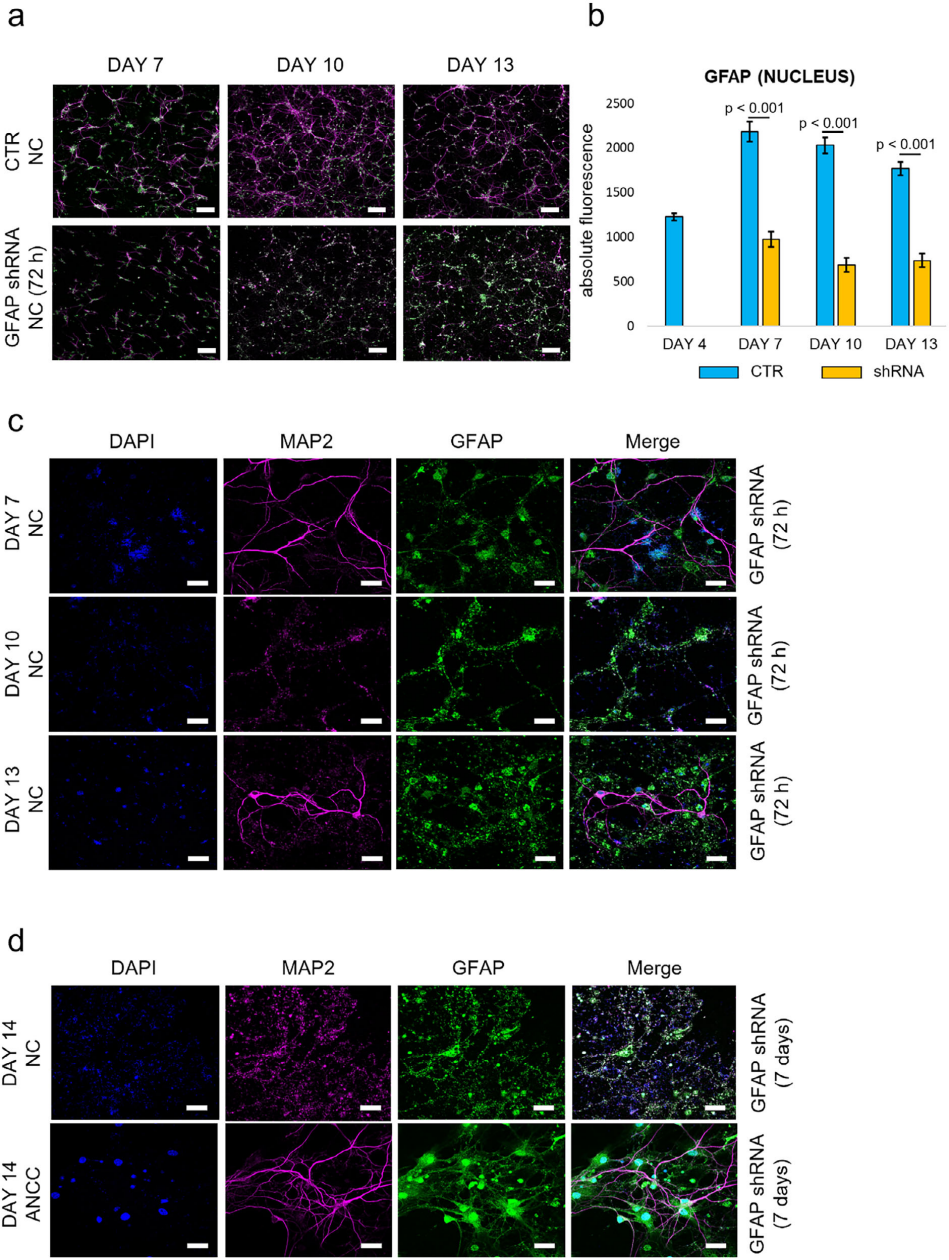
GFAP knockdown reduces GFAP‐associated fluorescence in neurons in a time‐dependent manner. (a) Representative immunofluorescence images of control neurons (CTR NC) and neurons transduced with GFAP‐targeting shRNA on days 4, 7, or 10 of in vitro culture. Cells were fixed 72 h after transduction, corresponding to days 7, 10, and 13, respectively. Scale bar = 100 µm. (b) Quantitative analysis of GFAP‐associated fluorescence intensity measured in neuronal nuclei. Data represent mean ± SEM from three independent experiments (*N* = 3, *n* = 3840 cells per culture time point). On days 7, 10, and 13 following 72 h of incubation with GFAP shRNA, statistically significant differences were observed compared to the control culture (p < 0.001). (c) Representative immunofluorescence images showing the effect of GFAP‐targeting shRNA on neuronal morphology and viability. GFAP‐associated signal was markedly reduced, accompanied by signs of nuclear disintegration (DAPI staining) and neurite fragmentation (MAP2 staining). Merged images illustrate loss of structural integrity and spatial disorganization of the neuronal network following GFAP knockdown. Scale bar = 20 µm. (d) Representative immunofluorescence images of neurons (NC) treated with GFAP shRNA show that, within 7 days, neuronal morphology and nuclear integrity are altered, in contrast to astrocyte‐neuron co‐cultures (ANCC), where GFAP knockdown does not affect neuronal morphology. Neurons were visualized using MAP2, nuclei were counterstained with DAPI, and GFAP expression is shown in green. Scale bar = 20 µm. For a), c), d) permeabilization with Triton X‐100 was performed for 45 min to enhance epitope accessibility.

Changes in GFAP expression over time during neuronal culture suggest that GFAP may play a role in neuronal development or maturation. To obtain preliminary information on whether GFAP is required for neuronal maturation—and simultaneously to verify that the GFAP expression observed in neurons is not a measurement artifact—we partially silenced GFAP expression using shRNA. The results showed that a 72‐h incubation of neurons with GFAP‐targeting shRNA significantly reduced GFAP‐associated fluorescent signal in neuronal cultures when the shRNA was applied on days 4 and 7 of culture, and to a slightly lesser extent when applied on day 10 (Figure 7a,b). No changes in GFAP was observed when cells were transduced with control lentivirus particles (Figure S2a).

The reduction of GFAP‐associated fluorescence by GFAP‐specific shRNA provides strong evidence that the observed changes in GFAP levels in neurons—detected using antibody‐based techniques—are not artifacts, but rather reflect genuine alterations in the protein's concentration. Subsequently to the shRNA‐mediated reduction in GFAP level, we observed significant alterations in morphology and viability of neurons (Figure 7c): nuclear structure appeared disintegrated (as shown by DAPI staining), and neurite morphology (visualized by MAP2 staining) was disrupted (Figure 7c). The most pronounced changes were observed when GFAP was silenced between 7 and 10 day of the culture growth while addition of anti GFAP shRNA for 72 h on day 10 were less deleterious for neurons. It suggests that GFAP is especially important for neurons during intense formation of new neurites.

Whatever the precise role of GFAP in neuronal maturation or development may be, the presence of this protein appears to be essential for neurons. This is evidenced by the fact that the addition of GFAP‐targeting shRNA for 3 days to 7‐day‐old neuronal cultures led to the death of nearly all cells (Figures 7d and S2b). Interestingly, a partial knockdown of GFAP expression in astrocyte‐neuron co‐cultures (at a 1:1 astrocyte‐to‐neuron ratio) did not significantly affect neuronal viability and morphology (Figures 7d and S2b). In contrast to pure neuronal cultures, incubation of the co‐culture with shRNA against GFAP for 7 days (added to the medium on day 7 of culturing) did not significantly affect cell morphology.

## 4 Discussion

Primary cultures of hippocampal neurons are widely used in neuroscience as a robust and reproducible in vitro model to study neuronal physiology, development, synaptic function, and disease mechanisms. Due to the critical role of hippocampus in learning, memory formation, and its vulnerability to neuropathological conditions, cultured hippocampal neurons provide a powerful system for investigating fundamental neuronal processes. Despite substantial advances in the application of proteomic techniques to brain tissue, a comprehensive, quantitative analysis of the in vitro hippocampal neuronal proteome has been lacking. This is in stark contrast to in‐depth proteomic studies of whole brain structures—including the hippocampus, cortex, and cerebellum—which have revealed significant age‐ and neurodegeneration‐dependent alterations in pathways related to synaptic plasticity, energy metabolism, and cytoskeletal dynamics [28, 53, 54, 64, 65, 66].

In this study, we presented the first global quantitative proteomic characterization of primary murine hippocampal neurons cultured for 14 days in vitro. Importantly, our cultures were maintained in medium containing 2.5 mM glucose—a concentration that closely reflects the concentration in brain interstitial fluid in vivo [67, 68]. Most previous studies have relied on supra‐physiological glucose concentrations (∼15‐25 mM), which may artificially shift metabolic pathways and induce transcriptional and functional alterations [69, 70]. High glucose can also lead to increased formation of advanced glycation end‐products (AGEs), disrupting membrane receptor and ion channel function [71, 72]. By using near‐physiological glucose conditions, we aimed to better recapitulate the native metabolic environment of neurons and reduce potential artifacts associated with high‐glucose culturing.

Quantitative proteomics using the TPA enabled absolute estimation of protein abundance across 14‐day‐old hippocampal neuronal cultures and age‐matched hippocampal tissue. We identified over 5,500 proteins in cultured neurons, reflecting the extensive molecular complexity of the neuronal proteome. Practically all of them were also found in hippocampal samples. As expected, principal component analysis revealed profound differences between the cultured neurons and hippocampal tissue, attributable in part to cellular composition—neuronal cultures were over 95% pure, while hippocampal tissue contained substantial nonneuronal populations [25].

The neuronal proteome displayed a metabolic signature consistent with reliance on oxidative phosphorylation: key enzymes of the TCA cycle and OXPHOS complexes were more abundant in neurons than in whole hippocampi. In contrast, glycolytic enzymes and glucose transporters –particularly those that are highly abundant in astrocytes—were less abundant in neurons.

This pattern aligns with the known division of roles in brain metabolism, where astrocytes predominantly metabolize glucose via glycolysis and provide lactate to neurons, which prefer oxidative routes [31, 73]. Interestingly, neurons also expressed gluconeogenic and glyconeogenic enzymes such as FBP2, PC, and PCK. While traditionally associated with astrocytes, their presence in neurons may reflect alternative roles, including redox balancing via the pentose phosphate pathway and modulation of synaptic plasticity via noncanonical mechanisms [35, 36].

To assess neuronal functionality under near‐physiological glucose conditions, we chemically induced LTP and LTD, canonical models of activity‐dependent plasticity. The associated phosphorylation of CAMK2 at S286 and activation of caspase‐3 confirmed that the cultured neurons retained the molecular machinery required for synaptic modulation. These results suggest that 2.5 mM glucose supports not only neuronal viability but also the expression of fundamental physiological responses.

However, proteome comparisons revealed that expression levels of major glutamatergic and GABAergic receptors, as well as intracellular signaling proteins such as CAMKs, MAPKs, PKA, and PKC, were significantly lower in cultured neurons than in hippocampal tissue. This underexpression may reflect an immature neuronal phenotype and a lack of glial‐mediated trophic and synaptogenic support, as observed in other in vitro systems [48, 74]. These findings underscore the need for caution when extrapolating functional data from pure neuronal cultures to more complex in vitro systems,

One of the most unexpected findings was the high abundance of GFAP in nominally pure neuronal cultures. Traditionally regarded as an astrocyte‐specific marker, GFAP was detected in neurons at levels comparable to metabolic enzymes and was confidently quantified via more than 40 peptides including 3 GFAP‐unique peptides in MS‐based proteomics. Notably, we independently confirmed these results by conducting a parallel experiment in a separate laboratory, using a separate neuronal culture. This independent analysis, based on quantitative proteomics, reproduced the elevated GFAP levels in neurons, thereby reinforcing the robustness and reproducibility of our original findings.

Both immunofluorescence and Western blot analyses confirmed GFAP expression in neurons and that its level—as compared to γ‐actin—was slightly higher than in astrocytes. However, there is a clear discrepancy between the GFAP levels quantified by mass spectrometry and the relative abundance detected through immunofluorescent and immunochemical methods. While the MS‐based quantitation appears reliable—given that the unexpectedly high expression involves only a single protein among thousands measured—several factors may lead to a significant underestimation of GFAP levels when assessed using immotechniques. These include reduced antibody affinity due to post‐translational modifications of neuronal GFAP, or limited epitope accessibility, for example as a result of tight, multicomponent complexes formed with other proteins and nucleic acids. Indeed, when we applied an extended 45‐min permeabilization protocol using Triton X‐100 – a reagent that both disrupts cellular membranes and other cellular component and destabilizes protein–protein interactions—we were able to observe a strong GFAP‐associated fluorescent signal in neurons.

Importantly, GFAP expression declined over time in culture and was virtually absent in neurons co‐cultured with astrocytes. Functional knockdown of GFAP in pure neuronal cultures using shRNA led to dramatic morphological changes and loss of neuronal viability, particularly when silencing was applied during early culture stages. Interestingly, the same silencing had no toxic effects in astrocyte‐neuron co‐cultures, where neurons did not express detectable levels of GFAP. These observations suggest that in the absence of astrocytes, neurons may upregulate GFAP as a compensatory mechanism to support cytoskeletal integrity or network formation. This hypothesis is supported by immunolocalization of GFAP in neurites and developing intercellular contacts. Notably, GFAP was also detected using IF technique in neuronal nuclei, suggesting an additional, potentially nuclear role. The latter observation was further corroborated by MS‐based quantification, which revealed that GFAP levels in neuronal nuclei were approximately four times higher than in the cytoplasm, suggesting a potentially critical role for GFAP in the regulation of nuclear processes—such as the maintenance of proper nuclear architecture—during neuronal maturation.

Although we are aware the neuronal expression of GFAP may be controversial, it has been reported under certain in vitro conditions and in response to stress [75], and in Alzheimer and Down syndrome pathology [76]. Our data strongly support the notion that GFAP is not a detection artifact but rather reflects an endogenous response to an astrocyte‐deprived environment or/and stress stimuli, such as the neuronal isolation procedure. The essential nature of GFAP for neuronal survival under these conditions raises the possibility of yet‐unknown functions of this protein beyond its classical role in astrocytes.

Our study underscores the value of label‐free proteomics in neuronal culture research. Unlike WB or IF, which suffer from limited linear dynamic range and antibody specificity issues, MS‐based proteomics provides highly reproducible, unbiased and quantitative insights into thousands of proteins simultaneously. The TPA method used here allows for absolute protein quantification, offering a robust foundation for functional and comparative studies.

Nevertheless, the neuronal cultures used in this study represent a simplified model system lacking the cellular heterogeneity and network activity of brain tissue. The lower expression of synaptic proteins in vitro, despite preserved responsiveness to LTP/LTD induction highlights the need to include glial support or organotypic models to more faithfully reflect brain physiology.

## 5 Conclusion

Our findings provide the first in‐depth proteomic profile of hippocampal neurons cultured under physiologically relevant glucose conditions. The data reveal metabolic and functional adaptations consistent with neuronal identity, while highlighting limitations associated with glia‐free culture systems. Notably, the unexpected expression and essential role of GFAP in neurons challenge conventional views of cell‐type specificity of this protein and point to unexplored mechanisms of cytoskeletal regulation and neuronal survival. By integrating high‐resolution proteomics with functional assays, this study sets a new benchmark for molecular characterization of neuronal cultures and opens avenues for investigating neuron‐glia interactions and adaptive responses in vitro.

## Author Contributions

D.D.‐F.—investigation, formal analysis, data curation, writing, visualization, original draft preparation; K.G.‐P.—investigation, formal analysis, writing, visualization; N.P.‐M.—investigation, formal analysis, writing, visualization; M.F.—investigation, formal analysis; A.G.—a investigation formal analysis, review and editing; J.R.W.—investigation, formal analysis, supervision; D.R.—formal analysis, original draft preparation, review and editing, supervision. All authors have read and agreed to the published version of the manuscript.

## Conflicts of Interest

The authors declare no conflicts of interest.

## Institutional Review Board Statement

All the procedures were approved by the local Ethical Commission (Wroclaw Ethical Committee, permission no.10/2018 and no. 041/2023) and every effort was made to minimize the number of animals used for the experiments.

## Supporting information




**Supporting File 1**: pmic70130‐sup‐0001‐TableS1.xlsx.

**Supporting File 2**: pmic70130‐sup‐0002‐TableS2.xlsx.

**Supporting File 3**: pmic70130‐sup‐0003‐TableS3.xlsx.

**Supporting File 4**: pmic70130‐sup‐0004‐Figures.docx.

## Data Availability

The data presented in this study are available on request from the corresponding authors. The data that support the findings of this study are openly available in ProteomeXchange Consortium (central repository PXD025978, files UU1‐UU5 for hippocampi of young murine) and in ProteomeXchange Consortium via the PRIDE (partner repository PXD038676 and PXD067465 for primary cultures of mice hippocampal neurons).
