# Supplementary material for: Proteomic Profiling of Primary Hippocampal Neurons Reveals Noncanonical GFAP Expression and Metabolic Adaptations in Glia‐Free Culture
Source: Proteomics. 2026 Apr 10;26(7):111–30. doi: 10.1002/pmic.70130 (PMC13327714; doi:10.1002/pmic.70130)
Supplement: Supplementary file 4 — Supporting File 4: pmic70130‐sup‐0004‐Figures.docx. [file PMIC-26--s003.docx]

**Supporting Information FIGURE S1**


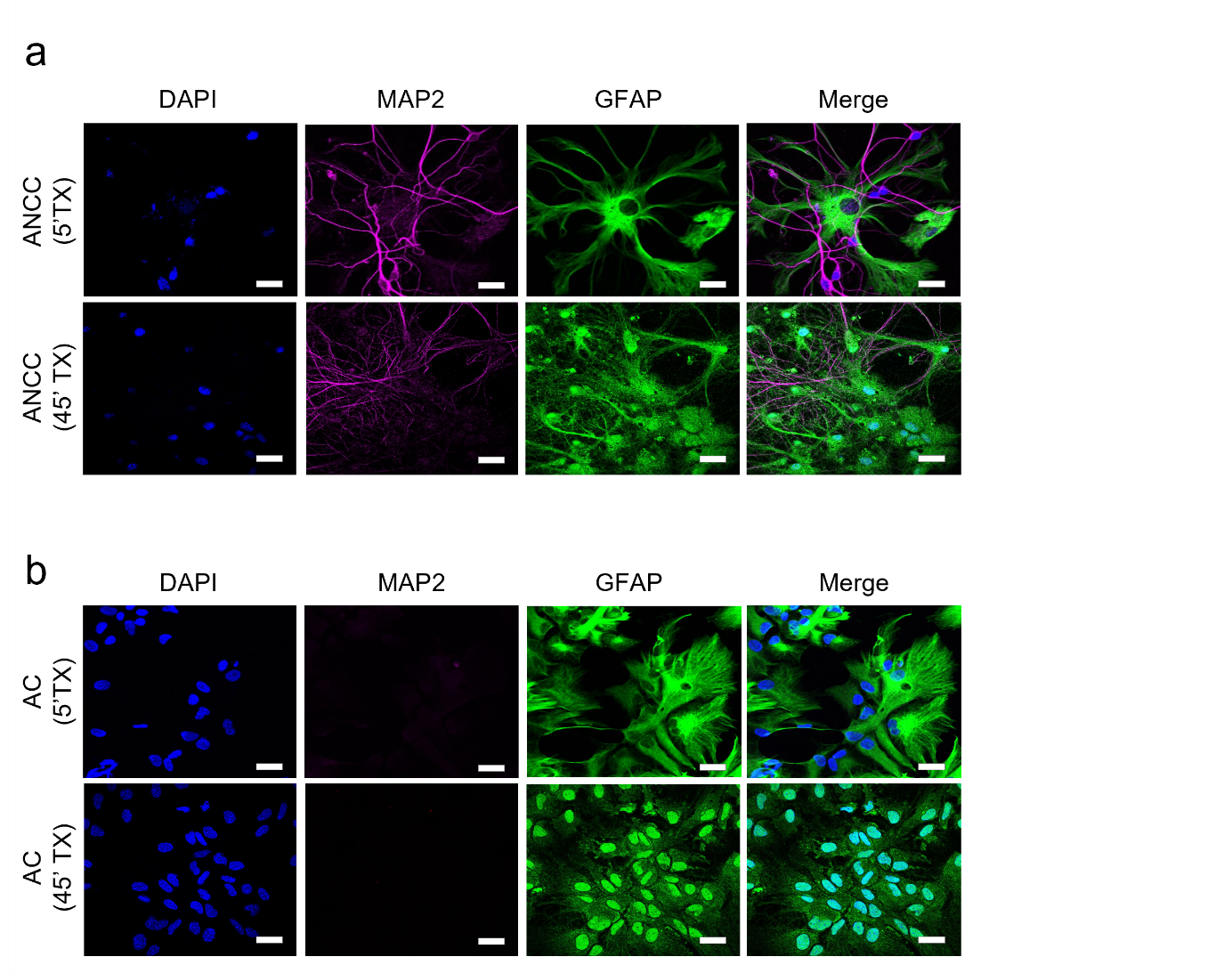


**Supporting Information FIGURE S1 Cell morphology in samples permeabilized with TRITON X-100 (TX) for 5 or 45 minutes.** (a) Cell morphology and GFAP- and MAP2-associated immunofluorescence signals in astrocyte–neuron co-cultures (ANCC). Scale bar = 20 µm. (b) Cell morphology and GFAP-associated signal in astrocyte monocultures (AC). Scale bar = 20 µm.

**Supporting Information FIGURE S2**


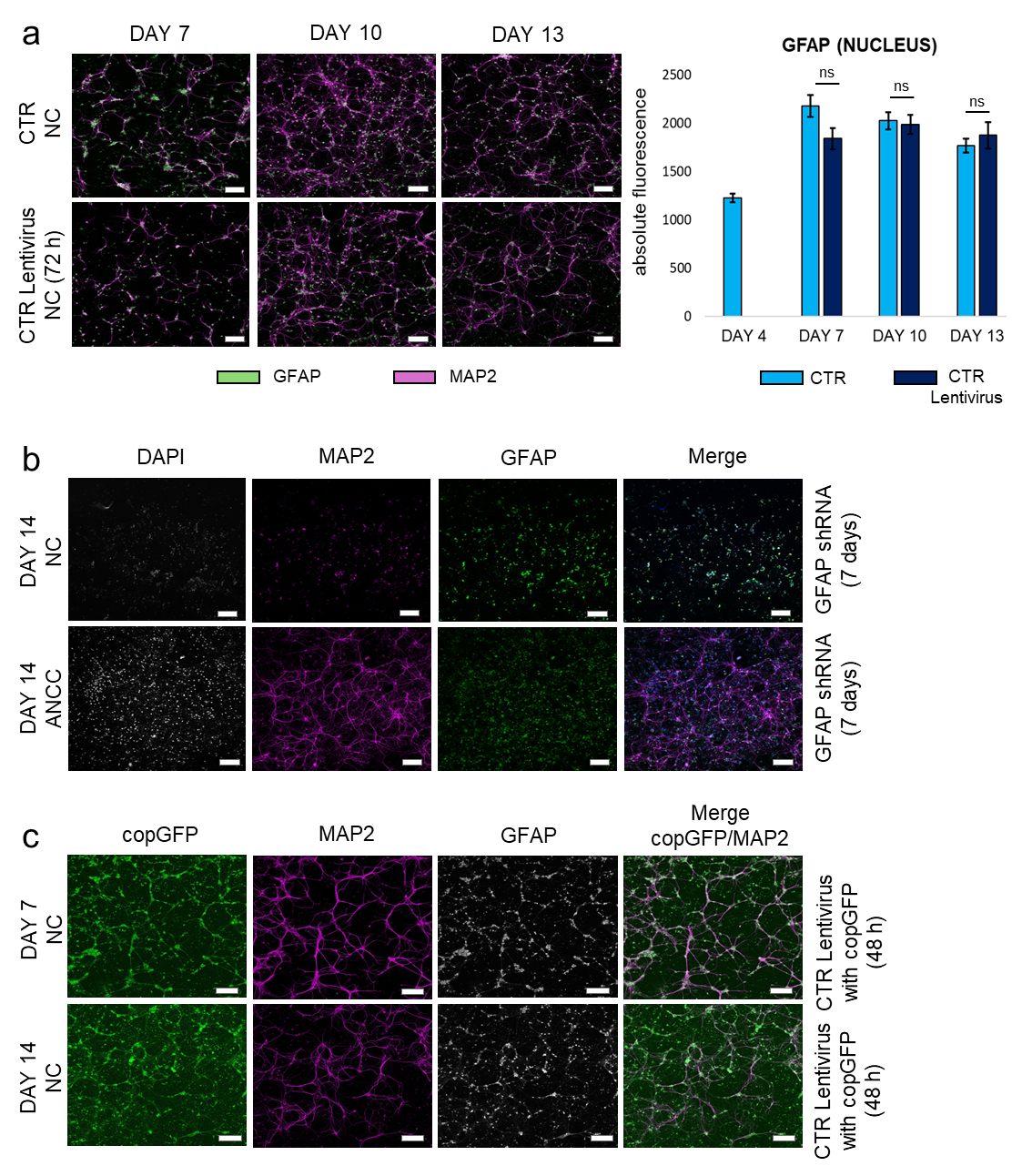


**Supporting Information FIGURE S2** (a) No changes in GFAP- or MAP2-associated signals were observed in neuronal monocultures (NC) following 72-hour incubation with the control lentivirus (CTR lentivirus). GFAP is shown in green (originally detected in the far-red channel using an Alexa Fluor 633–conjugated antibody, in order to avoid spectral overlap with the GFP-related signal from the control lentivirus expressing copGFP; see Materials and Methods and **Figure S2C**). Permeabilization with Triton X-100 was performed for 45 minutes to enhance epitope accessibility. Scale bar = 100 µm. Quantitative analysis of GFAP-associated fluorescence intensity within neuronal nuclei. Data are presented as mean ± SEM from three independent experiments. On days 7, 10, and 13 following 72-hour incubation with the lentivirus, no statistically significant differences were observed compared to the control culture. (b) The effect of a 7-day incubation of neuronal monocultures (NC) and astrocyte–neuron co-cultures (ANCC) with shRNA targeting GFAP, added to the medium on day 7 of culture. Permeabilization with Triton X-100 was performed for 45 minutes to enhance epitope accessibility. Scale bar = 100 µm. (c) Transduction efficiency was assessed using copGFP Control Lentiviral Particles (sc-108084, Santa Cruz). Fluorescence detection of copGFP at 48 hours post-transduction, analyzed on days 7 and 14 of neuronal culture (NC), indicated approximately 100% efficiency. Moreover, no changes in GFAP- or MAP2-associated signals were observed. Permeabilization with Triton X-100 was performed for 45 minutes to enhance epitope accessibility. Scale bar = 100 µm.
